# Morbidity and utilisation of healthcare services among people with cardiometabolic disease in three diverse regions of rural India

**DOI:** 10.1177/17423953231153550

**Published:** 2023-02-06

**Authors:** Sojib Bin Zaman, Roger G Evans, Clara K Chow, Rohina Joshi, Kavumpurathu R Thankappan, Brian Oldenburg, Ajay S Mahal, Kartik Kalyanram, Kamakshi Kartik, Michaela A Riddell, Oduru Suresh, Nihal Thomas, Gomathyamma K Mini, Pallab K Maulik, Velandai K Srikanth, Amanda G Thrift

**Affiliations:** 1 Department of Medicine, School of Clinical Sciences at Monash Health, 2541 Monash University, Melbourne, Australia; 2 Cardiovascular Disease Program, Biomedicine Discovery Institute and Department of Physiology, 2541 Monash University, Melbourne, Australia; 3 Pre-clinical Critical Care Unit, Florey Institute of Neuroscience and Mental Health, University of Melbourne, Melbourne, Australia; 4 George Institute for Global Health, 7800 University of New South Wales, Sydney, Australia; 5 Department of Cardiology, 8539 Westmead Hospital, Sydney, Australia; 6 Sydney Medical School, University of Sydney, Sydney, Australia; 7 George Institute for Global Health, New Delhi, India; 8 206426 Public Health and Community Medicine, Central University of Kerala, Kasaragod, India; 9 104284 Non-Communicable Diseases and Implementation Science, Baker Heart and Diabetes Institute, Melbourne, Australia; 10 Nossal Institute for Global Health, Melbourne School of Population and Global Health, University of Melbourne, Melbourne, Australia; 11 Rishi Valley Rural Health Centre, Chittoor District, India; 12 Department of Endocrinology, Diabetes & Metabolism, 30025 Christian Medical College, Vellore, India; 13 Global Institute of Public Health, 75682 Ananthapuri Hospitals and Research Institute, Trivandrum, India; 14 Peninsula Clinical School, Central Clinical School, 2541 Monash University, Frankston, Australia

**Keywords:** Cardiometabolic diseases, healthcare disparities, health insurance, private facilities, India

## Abstract

**Objectives:**

To assess the prevalence and determinants of cardiometabolic disease (CMD), and the factors associated with healthcare utilisation, among people with CMD.

**Methods:**

Using a cross-sectional design, 11,657 participants were recruited from randomly selected villages in 3 regions located in Kerala and Andhra Pradesh from 2014 to 2016. Multivariable logistic regression was used to identify factors independently associated with CMD and healthcare utilisation (public or private).

**Results:**

Thirty-four per cent (n = 3629) of participants reported having ≥1 CMD, including hypertension (21.6%), diabetes (11.6%), heart disease (5.0%) or chronic kidney disease (CKD) (1.6%). The prevalence of CMD was progressively greater in regions of greater socio-economic position (SEP), ranging from 19.1% to 40.9%. Among those with CMD 41% had sought any medical advice in the last month, with only 19% utilising public health facilities. Among people with CMD, those with health insurance utilised more healthcare (age-gender adjusted odds ratio (AOR) (95% confidence interval (CI)): 1.31 (1.13, 1.51)) as did those who reported accessing private rather than public health services (1.43 (1.23, 1.66)).

**Discussion:**

The prevalence of CMD is high in these regions of rural India and is positively associated with indices of SEP. The utilisation of outpatient health services, particularly public services, among those with CMD is low.

## Introduction

Cardiometabolic diseases (CMDs), including cardiovascular disease (CVD), diabetes and chronic kidney disease (CKD),^1^ are the leading cause of death worldwide.^2^ The prevalence of CMD is increasing in low- and middle-income countries (LMICs), including India.^3^ To develop programs to ameliorate the burden of CMD, policymakers require details about the factors associated with CMD and the barriers to their effective medical management. While there is relatively good information in India regarding the prevalence of specific CMDs and associated risk factors,^4–6^ and evidence that the prevalence of CVDs varies according to the level of epidemiological transition^7^ and wealth,^8^ less is known about the overall burden of CMD in rural settings and whether the burden differs by factors such as gender and socio-economic position (SEP).

Data on morbidity and utilisation of healthcare in India, captured periodically in national level surveys, including the National Sample Survey,^9^ indicate persistent inequalities in the use of outpatient and inpatient care in most states.^10^ However, our understanding of the degree to which the healthcare needs of those with CMD are met in rural India, the role of health insurance and the barriers to adequate healthcare utilisation, remains poor.

The primary objective of this study was to determine the prevalence of self-reported CMD and identify associated factors, with a focus on assessing the level of outpatient healthcare among people with CMD. The secondary objective was to determine the factors associated with utilisation of healthcare services.

We present the article in accordance with the Strengthening the Reporting of Observational Studies in Epidemiology (STROBE) reporting checklist (Supplemental Table S1).

## Methods

### Ethics and consent

This project was approved by the Health Ministry's Screening Committee of the Government of India (58/4/1F/CHR/2013/NCD II), the Sree Chitra Tirunal Institute of Medical Sciences and Technology (SCT/IEC-484/July 2013), the Centre for Chronic Disease Control (CCDC-IEC-09-2012), Christian Medical College Vellore and Monash University (CF13/2516-2013001327). Written informed consent was obtained from all participants prior to inclusion. When participants could not read or write, the patient information statement was read aloud to them and consent was recorded via a thumbprint.

### Study design and settings

We conducted a community-based observational study across three socioeconomically diverse regions in rural southern India, as described previously.^11^ In brief, using a cross-sectional design, we randomly selected villages from each region. Of the three regions, the northern part of the District of Trivandrum in Kerala (hereafter called Trivandrum) is the most socioeconomically advantaged and the Rishi Valley region in Southern Andhra Pradesh (hereafter called Rishi Valley) is the least. The West Godavari District in Northern Andhra Pradesh (hereafter called West Godavari) is intermediate between these two extremes. These three regions, at different points along the epidemiological transition, differ in terms of educational attainment, household income, type of employment, model of healthcare delivery and the role of health insurance (Supplemental Table S2).^12,13^

Using population censuses, either available through polling booth registers (Trivandrum) or explicitly conducted for this study (West Godavari and the Rishi Valley), a random subsample of 16,949 adult participants were invited to the survey. In total, 11,657 (response rate 69%) participants were recruited, with similar proportions of people in each age group and gender between sites (for further details, see Supplemental Table S2).

### Variables and their definitions

We defined people with CMD as those who self-reported CVD, diabetes mellitus or CKD at the time of interview. CVD included heart disease, hypertension, stroke, hypercholesterolaemia and coronary artery disease (CAD) including myocardial infarction (MI), coronary angioplasty or coronary artery bypass grafting (CABG). Recent healthcare utilisation was defined when participants self-reported visiting a medical care facility (public or private) for any of their needs at least once in the last month, as used by others previously.^14^ Variables, definitions and associated survey questions are listed in Supplemental Table S3.

### Data analytic approach

Utilisation of health services between the three study regions was compared using analysis of variance for continuous variables and chi-square for categorical variables. Univariable logistic regression was used to quantify the associations between the dichotomous outcome of CMD and the independent variables. Multivariable logistic regression was used to identify factors independently associated with utilisation of healthcare services among people with self-reported CMD, by all regions and stratified by individual regions. We incorporated covariates with a *p*-value of ≤0.2 from univariable logistic regression into the multivariable models.^15^ We also conducted sensitivity analyses to determine how target variables in the multivariable regression model were affected when adjusted for differing combinations of variables related to SEP (regions, education status, household income and ration card).^16^ We used the variance inflation factor (VIF) for assessing multicollinearity between variables. We also identified the most suitable model using the Akaike information criteria (AIC) and Bayesian information criteria (BIC). The same approach was used to identify factors independently associated with the choice of healthcare providers (private vs. public) among people with CMD. All analyses were conducted using Stata 15 (StataCorp, College Station, Texas, USA). Two-sided *p* ≤ 0.05 was considered statistically significant.

## Results

### Demographic characteristics

The participants’ mean age was 45.5 years and 50.3% were women (Table 1). SEP as assessed by literacy, educational attainment, type of ration card and household income, was greatest in Trivandrum and least in the Rishi Valley. This gradient of greater SEP was associated with a progressively greater proportion of people with a waist circumference (WC) above normal and self-reported alcohol consumption. In contrast, other risk factors, such as current smoking and physical inactivity, while varying between regions, did not vary progressively with indices of SEP. Self-reported difficulty in accessing healthcare was progressively less in areas of greater SEP. Use of public healthcare providers was greatest in Trivandrum (57.9%), least in West Godavari (6.0%) and intermediate in the Rishi Valley (19.2%). Participants were least likely to have health insurance in Trivandrum (54.7%), but their tendency to have sought medical advice in the last 1 month (31.2%) or 3 months (49.8%) was greater than in the other two regions. In the month prior to interview, women (29.4%) utilised healthcare more than men (24.3%).

**Table 1.** Sociodemographic characteristics of all participants, by gender and region.

Characteristics	Stratified by gender			All participants (11,657)	Stratified by region			
	Men (n = 5784)	Women (n = 5852)	*P_Gender_*		Rishi Valley (3400)	West Godavari (4500)	Trivandrum (3757)	*P*_Region_
*Demographic*								
Women^c^				5852 (50.3)	1700 (50.0)	2248 (50.1)	1904 (50.6)	0.835
Age [mean, SD] year	45.5 [17.4]	45.4 [17.0]	0.096	45.5 [17.2]	45.9 [16.6]	44.7 [17.3]	46.0 [17.7]	0.001
Age group			0.245					0.003
<50 years	3290 (56.8)	3391 (57.9)		6693 (57.4)	1932 (56.8)	2637 (58.6)	2124 (56.5)	
≥50 years	2494 (43.1)	2461 (42.1)		4964 (42.6)	1468 (43.2)	1863 (41.4)	1633 (43.5)	
Marital status			<0.001					<0.001
Never married	1108 (19.3)	361 (6.2)		1472 (12.6)	286 (8.5)	507 (11.2)	679 (18.0)	
Married	4426 (76.8)	4084 (70.1)		8524 (73.4)	2595 (77.3)	3322 (73.8)	2607 (69.3)	
Separated/ divorced/ widow/er	226 (3.9)	1382 (23.7)		1612 (13.8)	472 (14.0)	669 (14.8)	471 (12.5)	
*Socio-economic position*								
Literate	4139 (71.9)	3287 (56.4)	<0.001	7436 (64.0)	1537 (45.8)	2527 (56.1)	3372 (89.7)	<0.001
Educational attainment^d^			<0.001					<0.001
No formal schooling	917 (16.2)	1847 (32.2)		2769 (24.3)	1162 (36.7)	1209 (26.9)	398 (10.7)	
≤ Class 6 completed	1546 (27.3)	1406 (24.5)		2959 (25.9)	793 (25.1)	1650 (36.8)	516 (13.7)	
7 −10 completed	1982 (35.1)	1628 (28.4)		3616 (31.7)	830 (26.2)	1100 (24.5)	1686 (44.8)	
≥ Class 12 completed	1208 (21.4)	849 (14.9)		2060 (18.1)	380 (12.0)	523 (11.8)	1157 (30.8)	
Household income^e^			<0.001					<0.001
Rs. 0 to 1000 (Q1)	1413 (24.4)	1729 (29.5)		3149 (27.0)	1851 (54.4)	514 (11.4)	784 (20.9)	
Rs. > 1000–1900, Q2	1228 (21.2)	898 (15.4)		2130 (18.3)	561 (16.5)	1042 (23.2)	527 (14.0)	
Rs. > 1900–3000, Q3	1375 (23.8)	1186 (20.3)		2565 (22.0)	446 (13.1)	1646 (36.6)	473 (12.6)	
Rs. > 3000, Q4	1129 (19.5)	1097 (18.7)		2231 (19.1)	490 (14.5)	1135 (25.2)	606 (16.1)	
Missing group, Q0	639 (11.1)	942 (16.1)		1582 (13.6)	52 (1.5)	163 (3.6)	1367 (36.4)	
Type of ration card^c^			<0.001					<0.001
No ration/APL	1393 (24.2)	1537 (26.4)		2932 (25.3)	186 (5.6)	444 (9.8)	2302 (61.3)	
BPL	4267 (74.2)	4103 (70.4)		8389 (72.3)	2888 (86.3)	4050 (90.1)	1451 (38.6)	
Poorest of the poor	94 (1.6)	184 (3.2)		278 (2.4)	272 (8.1)	4 (0.1)	2 (0.1)	
*Risk factors*								
Current smoker^c^	1706 (29.7)	100 (1.7)	<0.001	1813 (15.6)	450 (13.4)	779 (17.3)	584 (15.5)	<0.001
Ever taken alcohol^d^	2401 (42.6)	56 (0.9)	<0.001	2465 (21.6)	510 (16.2)	955 (21.2)	1000 (26.6)	<0.001
Physical activity^a,d^	4264 (79.5)	4589 (84.5)	<0.001	8871 (82.0)	1825 (71.1)	4260 (94.6)	2786 (74.2)	<0.001
WC above normal^b^	1752 (30.4)	2719 (46.8)	<0.001	4535 (38.9)	665 (19.5)	1932 (43.1)	1938 (51.5)	<0.001
*Health care utilisation*								
Healthcare sought in the last 3 months^d^	2075 (38.0)	2603 (47.1)	<0.001	4682 (42.5)	1205 (43.8)	1603 (35.6)	1874 (49.8)	<0.001
Healthcare sought in the last month^c^	1403 (24.3)	1717 (29.4)	<0.001	3123 (26.9)	699 (20.8)	1252 (27.8)	1172 (31.2)	<0.001
Health insurance^d^	4107 (71.5)	4198 (71.7)	0.417	8320 (71.9)	2753 (82.1)	3512 (78.7)	2055 (54.7)	<0.001
Government insurance	4031 (69.7)	4130 (70.6)	0.299	8176 (70.1)	2749 (80.8)	3531 (78.5)	1896 (50.5)	<0.001
Private insurance	67 (1.2)	73 (1.2)	0.659	140 (1.2)	8 (0.2)	10 (0.2)	122 (3.3)	<0.001
Employer insurance	65 (1.1)	62 (1.1)	0.738	127 (1.1)	7 (0.2)	10 (0.2)	110 (2.9)	<0.001
NGO insurance	7 (0.1)	6 (0.1)	0.765	13 (0.1)	7 (0.2)	6 (0.1)	0 (0)	0.029
Self-reported difficulty in getting to healthcare^c^			<0.001					<0.001
Easy	4447 (77.2)	4055 (69.6)		8516 (73.4)	1903 (56.8)	3187 (70.9)	3426 (91.2)	
Difficult	1312 (22.8)	1768 (30.4)		3087 (26.6)	1449 (43.2)	1307 (29.1)	331 (8.8)	
Type of healthcare usually sought^d^			0.241					<0.001
Private	3873 (72.3)	3976 (73.3)		7868 (72.8)	2061 (80.8)	4225 (93.9)	1582 (42.1)	
Public	1484 (27.7)	1448 (26.7)		2934 (27.2)	489 (19.2)	270 (6.1)	2175 (57.9)	

**^a^** Participants were considered physically active if they had undertaken any physical activity for more than 30 minutes at least five times a week or had undertaken vigorous physical activity at least three times per week.

^b^ Waist circumference above normal was defined as >90 cm for men and >80 cm for women.

^c^ 21–88 missing observations.

^d^ 253–855 missing observations.

^e^ 1582 missing observations.

Data are presented as proportion (%) or mean with standard deviation (SD).

*P*-values for differences between gender and regions were generated using one-way ANOVA (for continuous variables) or the chi-square test (for categorical variables).

Smoker referred to those smoking tobacco products and did not include smokeless tobacco products.

The categories for types of health insurance do not sum to the overall number (%) of people with health insurance because some people have more than one type of health insurance.

APL, Above Poverty Line; BPL, Below Poverty Line; NGO, non-governmental organisation; Q, quartile; WC, waist circumference.

### Profile of morbidity

#### Prevalence of self-reported CMD

At the time of survey administration, 3629 (34.1%) reported at least one CMD (Table 2), such as hypertension (21.6%), diabetes (11.6%), hypercholesterolaemia (7.5%), heart disease (5.0%), stroke (1.8%) and CKD (1.6%).

**Table 2.** Prevalence of self-reported cardiometabolic disease (CMD) and risk factors (n =  11,657), stratified by gender and region.

Characteristics	Stratified by gender			All sites combined (11,657)	*Stratified by region*			
	Men (n = 5784)	Women (n = 5852)	*P_Gender_*		Rishi Valley (3400)	West Godavari (4500)	Trivandrum (3757)	*P_Region_*
*CMDs*			** **					** **
Heart disease^b^			0.135					<0.001
Yes	292 (5.4)	298 (5.6)		590 (5.0)	87 (3.2)	248 (5.5)	255 (6.8)	
No	5048 (93.4)	5124 (93.0)		10,191 (87.4)	2483 (91.9)	4227 (94.4)	3481 (92.8)	
Don’t know	63 (1.2)	89 (1.4)		152 (1.3)	131 (4.9)	5.54 (0.1)	17 (0.4)	
Stroke^a^			<0.001					<0.001
Yes	139 (2.4)	75 (1.3)		215 (1.8)	40 (1.2)	116 (2.6)	59 (1.6)	
No	5569 (96.8)	5685 (97.6)		11,274 (97.1)	3209 (95.7)	4377 (97.3)	3688 (98.2)	
Don’t know	48 (0.8)	68 (1.1)		116 (1.1)	104 (3.1)	3 (0.1)	9 (0.2)	
Diabetes^a^			0.885					<0.001
Yes	666 (11.6)	669 (11.5)		1339 (11.6)	174 (5.2)	534 (11.8)	631 (16.8)	
No	4978 (86.5)	4971 (85.3)		9966 (85.8)	3067 (91.4)	3873 (86.2)	3026 (80.5)	
Don’t know	114 (1.9)	187 (3.2)		301 (2.6)	113 (3.4)	88 (2.0)	100 (2.7)	
CKD^b^			<0.001					<0.001
Yes	123 (2.3)	56 (1.0)		179 (1.6)	64 (2.5)	52 (1.2)	63 (1.7)	
No	5203 (96.9)	5356 (98.5)		10,580 (97.8)	2499 (97.3)	4430 (98.4)	3651 (97.2)	
Don’t know	41 (0.8)	24 (0.5)		65 (0.6)	6 (0.2)	16 (0.4)	43 (1.1)	
*Risk Factors*								
Hypertension^a^			<0.001					<0.001
Yes	990 (17.2)	1505 (25.8)		2501 (21.6)	401 (11.9)	1058 (23.5)	1042 (27.7)	
No	4757 (82.5)	4309 (73.9)		9081 (78.2)	2942 (87.8)	3435 (76.4)	2704 (71.9)	
Don’t know	15 (0.3)	13 (0.3)		28 (0.2)	11 (0.3)	6 (0.1)	11 (0.4)	
Hypercholesterolaemia^a^			<0.001					<0.001
Yes	344 (5.9)	525 (9.1)		870 (7.5)	32 (0.9)	93 (2.1)	745 (19.8)	
No	5213 (90.6)	5055 (86.7)		10,288 (88.6)	3200 (95.4)	4239 (94.3)	2849 (75.8)	
Don’t know	204 (3.5)	247 (4.2)		451 (3.9)	122 (3.7)	166 (3.6)	163 (4.4)	
Number of CMD			<0.001					<0.001
None reported	4172 (72.1)	3841 (65.6)		8028 (68.9)	2751 (80.9)	3057 (67.9)	2220 (59.1)	
One	943 (16.3)	1188 (20.3)		2133 (18.2)	509 (14.9)	913 (20.3)	711 (18.9)	
Two	373 (6.5)	532 (9.1)		907 (7.8)	104 (3.1)	374 (8.3)	429 (11.4)	
≥Three	296 (5.1)	291 (5.0)		589 (5.1)	36 (1.1)	156 (3.5)	397 (10.6)	
Reporting at least one CMD or risk factor	1612 (30.5)	2011 (37.6)	<0.001	3629 (34.1)	649 (24.2)	1443 (33.3)	1537 (42.4)	<0.001

^a^ 41–67 missing observations.

^b^ 724–833 missing observations.

Data are presented as proportion (%).

*P*-values for differences according to gender and three regions were generated using chi-square tests.

CMDs include heart disease, stroke, diabetes and CKD.

There were also 21 missing observations on gender, so these individuals were not included in the analysis.

CKD, chronic kidney disease.

More women reported having at least one CMD (34.4%) than men (27.9%) (Table 2). In addition, women more commonly reported risk factors such as hypertension and hypercholesterolemia than men. However, the prevalence of self-reported heart disease and diabetes did not vary significantly by gender.

When stratifying the burden of CMD by region (Table 2) the prevalence of heart disease and diabetes, but not stroke or CKD, was progressively greater in regions of higher SEP. Furthermore, the prevalence of co-existence of two or more CMDs was also progressively greater in regions of higher SEP.

The prevalence of hypertension was 29.7%, defined according to the measurement of blood pressure and self-reported use of antihypertensive medications, and was greater than the proportion self-reporting hypertension (21.6%; Supplemental Table S4). This apparent deficit in awareness and treatment of hypertension was more prominent in regions of lesser, than greater, SEP, as identified previously in these populations.^13^

#### Factors associated with self-reported CMD

**Figure 1.**
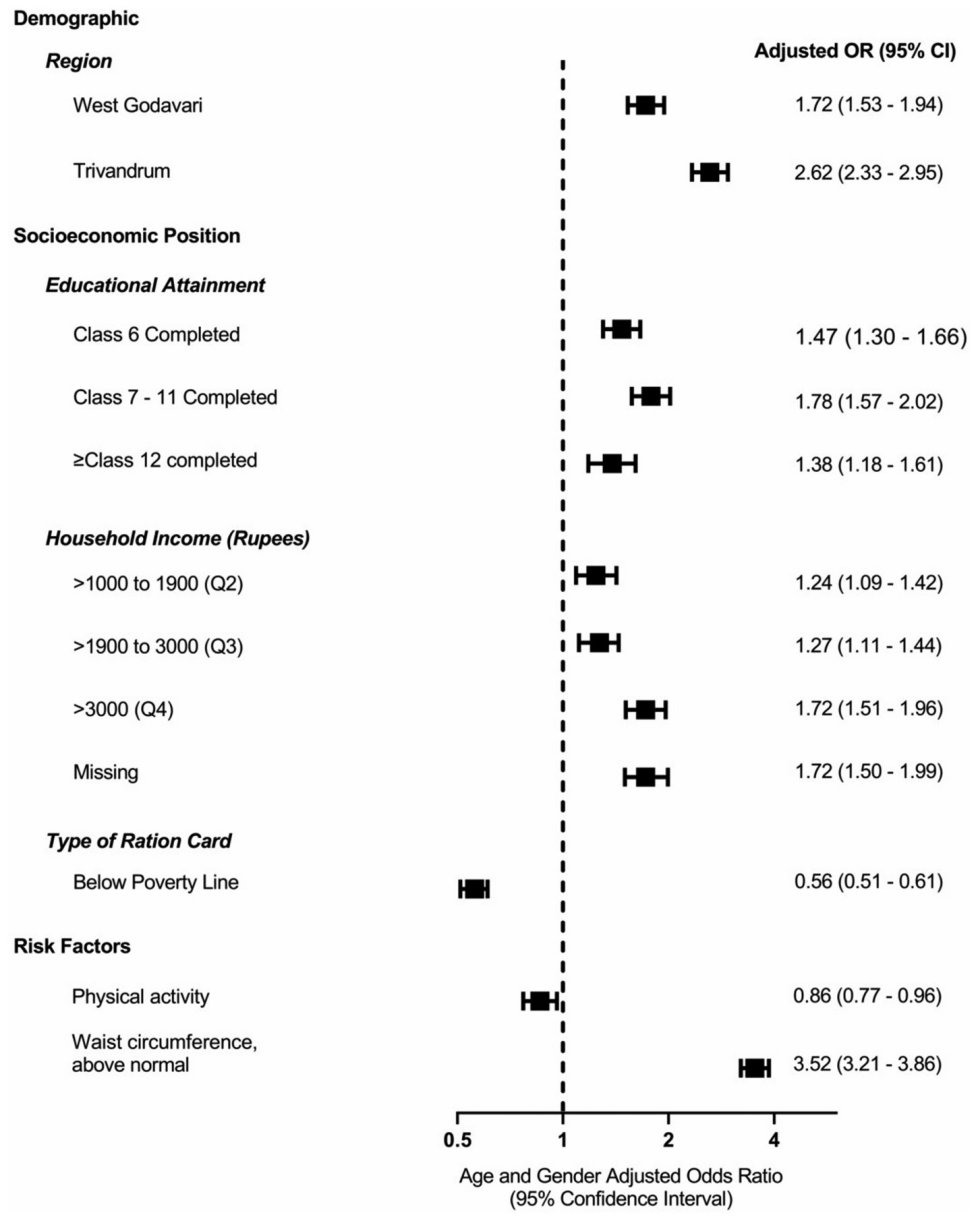
Factors associated with cardiometabolic diseases (CMDs) across all sites: multivariable logistic regression adjusted by age and gender. Data are presented as odds ratios (OR) with 95% confidence intervals (CI). Participants were considered physically active if they had undertaken any physical activity for more than 30 minutes at least five times a week or had undertaken vigorous physical activity at least three times per week. Waist circumference above normal was defined as >90 cm for men and >80 cm for women. There were 843 missing observations on income. Each variable is individually adjusted with age and gender.

After adjusting for age and sex, larger household income was positively associated with CMD (Figure 1). Additionally, people with lower SEP, defined according to the use of a Below Poverty Line (BPL) ration card, were less likely to report having a CMD than those who did not have a BPL ration card.

In multivariable logistic regression, the odds of CMD was 4.65-fold greater in those aged ≥50 years than those who were younger, 1.26-fold greater in women than men, progressively greater in the regions of greater SEP, West Godavari (1.77-fold) and Trivandrum (2.55-fold) than the Rishi Valley and 3.15-fold greater in those with a WC above normal (Model 6, Supplemental Table S5). For the most part, these patterns were also evident in other models (Model 1 to Model 5) that were adjusted for various combinations of confounding factors (Supplemental Table S5).

### Healthcare utilisation among people with CMD

#### Pattern of health service utilisation

In total, 41.3% of people with CMD had sought medical advice in the last month and 60.7% in the previous 3 months (Supplemental Table S6). Women (43.2%) had more commonly sought medical advice for managing their CMD in the last month than men (39.0%), but also reported more difficulty (29.5%) in accessing healthcare (23.0%). Health services were predominantly sought from private hospitals or clinics (41.4%) rather than public facilities (19.1%) such as government/public hospitals, primary healthcare centres or mobile clinics.

Overall, more people with CMD sought care from a physician and/or specialised doctor (31.6%) than Rural Medical Practitioners (RMP) (6%), Community Nurses (0.4%) or Accredited Social Health Activists (ASHAs) (0.3%) (Supplemental Table S6). Among people with CMD, only 58.6% reported taking any medications. Those with self-reported CMD commonly had at least one kind of health insurance (69.2%), predominantly government health insurance (95.6%), which does not cover expenditure incurred in private facilities or outpatient visits. Participants also relied on savings (37.4%), assistance and gifts (3.6%) and unsecured loans (2.1%) to cover expenditure for health care.

#### Factors associated with any healthcare utilisation in the previous month among people with CMD

**Figure 2.**
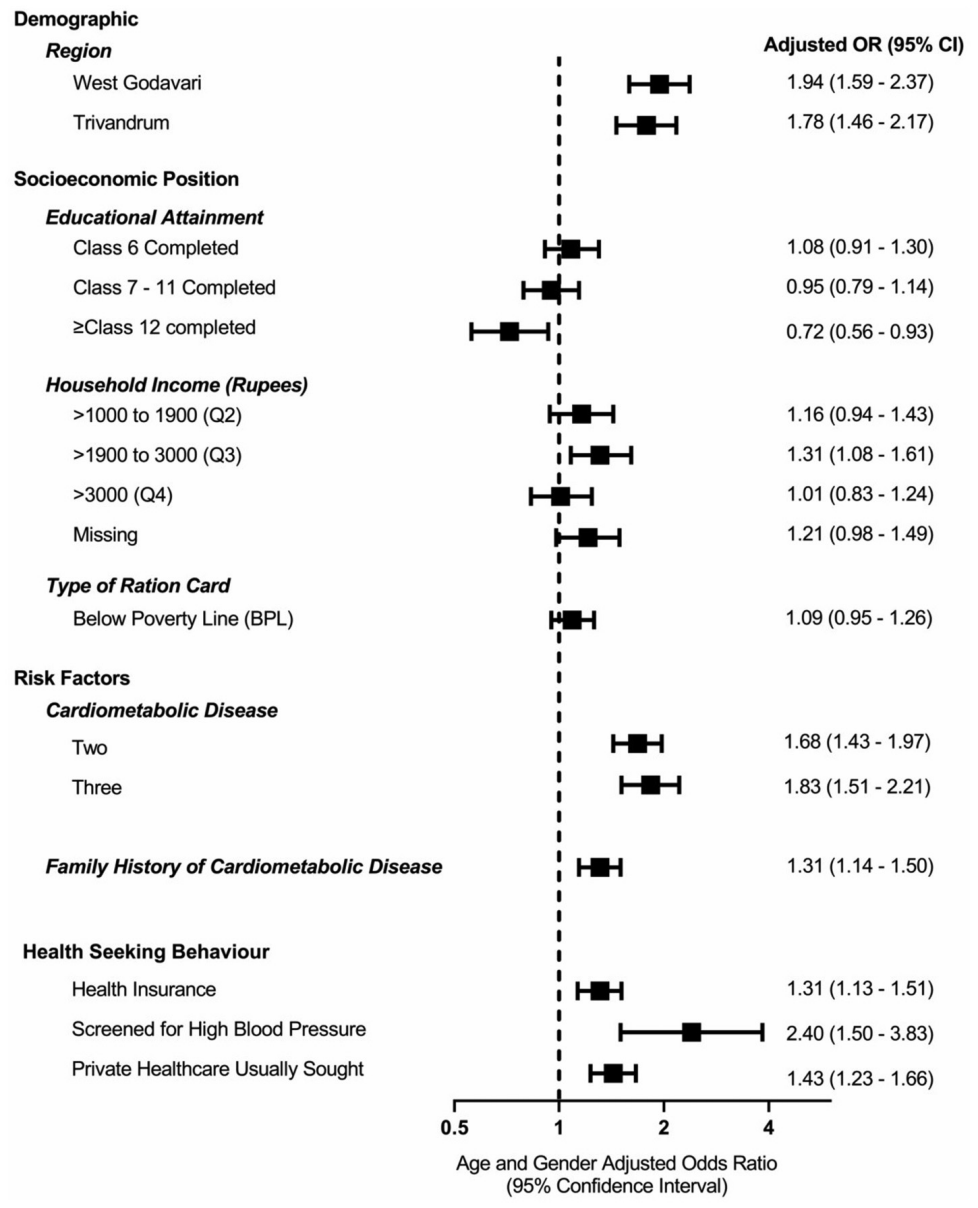
Factors associated with utilisation of healthcare providers among people with self-reported cardiometabolic diseases (CMDs) across all sites: multivariable logistic regression adjusted for age and gender. Data are presented as odds ratios (OR) with 95% confidence intervals (CI). There were 630 missing observations on income. Each variable is individually adjusted with age and gender.

After adjusting for age and sex, among people with CMD, those with health insurance utilised more healthcare (1.31-fold) as did those who reported accessing private rather than public health services (1.43-fold) (Figure 2).

In multivariable logistic regression, residing in a region other than the Rishi Valley (the region of least SEP) was associated with utilisation of healthcare, but individual measures of SEP (education, household income and ration card) were not significantly associated with healthcare utilisation (Model 6, Supplemental Table S7). There was also no evidence that individual measures of SEP were associated with any healthcare utilisation after stratifying by separate regions (Supplemental Tables S8 to S10). Interestingly, the association between self-reported medication use and healthcare utilisation was similar across the various bands of educational attainment (Supplemental Table S11). Yet, if healthcare utilisation was limited to that provided by public health providers, there was an inverse relationship between individual-level SEP and the use of public healthcare (Table 3) but not for private providers (Supplemental Table S12).

**Table 3.** Factors associated with utilisation of public healthcare providers among people with self-reported cardiometabolic diseases (CMDs).

Characteristics	Health care providers		Univariable analysis	Model 1 (each variable is adjusted for age and gender)	Model 2 (adjusted for age, gender and region)	Multivariable model (each variable is adjusted for all other variables in this column)
	Private (n = 2490)	Public (n = 989)	OR (95% CI)	AOR (95% CI)	AOR (95% CI)	AOR (95% CI)
Age group						
<50 years	785 (31.5)	332 (33.5)	Ref	─	─	Ref
≥50 years	1705 (68.5)	657 (66.5)	0.91 (0.78, 1.07)			0.81 (0.65, 0.99)*
Gender^a^						
Men	1089 (43.8)	440 (44.5)	Ref			Ref
Women	1395 (56.2)	549 (55.5)	0.97 (0.84, 1.13)	─	─	0.75 (0.62, 0.91)***
Region						
Rishi Valley	410 (16.4)	90 (9.1)	Ref	Ref	─	Ref
West Godavari	1357 (54.5)	85 (8.6)	0.29 (0.21, 0.39)***	0.29 (0.21, 0.4)***	─	0.38 (0.27, 0.53)***
Trivandrum	723 (29.1)	814 (82.3)	5.13 (4.00, 6.58)***	5.22 (4.07, 6.71)***	─	15.66 (11.23, 21.84)***
Education^b^						
No schooling	700 (28.2)	224 (22.6)	Ref	Ref	Ref	Ref
≤ Class 6 completed	799 (32.2)	231 (23.4)	0.90 (0.73, 1.11)	0.92 (0.74, 1.14)	0.76 (0.58, 0.98)*	0.82 (0.63, 1.07)
7 −11 completed	699 (28.1)	403 (40.8)	1.80 (1.48, 2.19)***	1.86 (1.52, 2.28)***	0.51 (0.39, 0.65)***	0.70 (0.53, 0.92)**
≥Class 12	286 (11.5)	130 (13.2)	1.42 (1.10, 1.84)**	1.50 (1.14, 1.96)***	0.34 (0.24, 0.47)***	0.55 (0.38, 0.78)***
Household income^c^						
Rs. 0 to 1000, Q1	535 (21.5)	282 (28.5)	Ref	Ref	Ref	Ref
Rs. > 1000–1900, Q2	482 (19.4)	138 (13.9)	0.54 (0.43, 0.69)***	0.52 (0.41, 0.66)***	0.59 (0.44, 0.78)***	0.64 (0.48, 0.86)***
Rs. > 1900–3000, Q3	578 (23.2)	131 (13.3)	0.43 (0.34, 0.55)***	0.41 (0.33, 0.53)***	0.65 (0.49, 0.87)***	0.71 (0.52, 0.96)**
Rs. > 3000, Q4	575 (23.1)	128 (12.9)	0.42 (0.33, 0.54)***	0.41 (0.32, 0.52)***	0.42 (0.32, 0.56)***	0.62 (0.46, 0.84)***
Missing group, Q0	320 (12.8)	310 (31.3)	1.83 (1.48, 2.27)***	1.90 (1.53, 2.35)***	0.77 (0.59, 0.99)*	0.88 (0.68, 1.15)
Type of ration card^a^						
APL or none	771 (30.9)	445 (44.9)	Ref	Ref	Ref	Ref
BPL	1718 (69.1)	544 (55.1)	0.55 (0.47, 0.63)***	0.55 (0.47, 0.64)***	3.03 (2.44, 3.76)***	2.16 (1.71, 2.73)***
CMD						
One	1479 (59.4)	533 (53.8)	Ref	Ref	Ref	Ref
Two	626 (25.1)	255 (25.7)	1.13 (0.95, 1.35)	1.16 (0.97, 1.39)	0.84 (0.68, 1.04)	0.79 (0.63, 0.98)*
≥Three	385 (15.5)	201 (20.5)	1.45 (1.19, 1.76)***	1.52 (1.24, 1.86)***	0.72 (0.56, 0.89)***	0.68 (0.53, 0.88)***
Health insurance^a^						
No	818 (33.1)	275 (27.8)	Ref	Ref	Ref	Ref
Yes	1657 (66.9)	713 (72.2)	1.28 (1.09, 1.51)**	1.27 (1.09, 1.50)***	2.83 (2.33, 3.44)***	2.35 (1.92, 2.88)***

^a^ 2–16 missing observations.

^b^ 63 missing observations.

^c^ 630 missing observations.

There are also 150 missing observations on health care providers, so these individuals were not included in the analysis.

Data are presented as the proportion (%) or odds ratios (95% confidence interval). There are 630 missing observations on household income (hereafter referred to as ‘Missing’, Q0), and these are included in the multivariable logistic regression.

Model 1: each variable is individually adjusted with age and gender.

Model 2: each variable is individually adjusted for age, gender and region.

The multivariable model is adjusted for age, gender, region, educational attainment, household income, ration type, CMD and health insurance (everything is adjusted for all).

*p < 0.05; **p < 0.01; ***p < 0.001.

AOR, adjusted odds ratio; APL, Above Poverty Line; BPL, Below Poverty Line; CI, confidence interval; OR, odds ratio; Q, quartile; Ref, reference category.

The use of public healthcare providers was more likely in people residing in Trivandrum (the region of greatest SEP; 15.66-fold), but less likely in those residing in West Godavari (0.38-fold) than in the Rishi Valley (Table 3), a pattern consistently observed after adjustment for all combinations of the various possible confounding factors. Additionally, utilisation of public healthcare rather than private was greater among those living below the poverty line (2.16-fold), and in those who had health insurance (2.35-fold) (Supplemental Table S13). However, these patterns were less marked in the Rishi Valley than in the other regions (Supplemental Table S14).

## Discussion

### Main findings

We found a large burden of CMD across three diverse populations in rural India which was greater in women than men. Risk of CMD is exacerbated by affluence but may be mitigated by education. There is a considerable treatment gap among people with CMD. Higher educational attainment was not associated with greater service utilisation but was associated with the use of private healthcare services. Major barriers to uptake of any healthcare services appear to include lack of government-issued health insurance, lack of awareness of risk factors and less utilisation of public healthcare facilities. However, more context-specific factors may contribute, with access to public healthcare being greater in Trivandrum, within the state of Kerala, than the two sites within Andhra Pradesh, even after adjustment for individual differences in socio-economic factors. State-based health systems may affect the accessibility to public healthcare services and thus healthcare services in general.

Although 31.1% of surveyed participants reported having at least one CMD, this likely underestimates the true prevalence of these CMDs, since many individuals may be unaware of their condition(s). This conclusion is supported by our finding that self-reported hypertension (21.6%) was considerably less than the prevalence of hypertension measured by standard diagnostic criteria (29.7%).

CMD was greater in people living in households with higher income, confirming that chronic diseases such as CMDs are ‘diseases of affluence’ in some LMICs.^17,18^ However, it could also be that people with greater educational attainment are more often screened for risk factors and have greater knowledge about these risk factors than less educated people.^19^ We also observed an association of progressively greater prevalence of CMD with the overall gradient of SEP across the three regions, which may be partly attributable to greater screening. However, we found that people with higher educational attainment were less likely to report having a CMD than people with no formal education. Thus, greater education might decrease the likelihood of CMD by motivating people to modify risk factors, although this does not appear to translate to better utilisation of healthcare (see below).

Only 41.3% of those with CMD had accessed healthcare in the previous month, indicating a treatment gap in rural India. The factors associated with healthcare utilisation, such as SEP, self-report of healthcare being easy to access and health insurance, are mostly modifiable. We also found relatively little utilisation of healthcare services from RMPs, Community Nurses and ASHAs (<7%) compared with doctors (30%) alone. This could indicate that these primary healthcare providers are under-utilised compared with medical doctors, at least with regard to CMD.

Accessibility of healthcare may be another factor influencing healthcare utilisation. Women reported more difficulty in accessing healthcare than men, in accordance with previous findings in a high income country.^20^ Real or perceived inaccessibility of healthcare might negatively influence decision-making about utilising health services.^21^ As reported by others, lack of trust in the public health system may also contribute,^22^ a factor which may explain why people do not access primary health centres even though they are almost free.^23^ Such erosion of trust can primarily occur at the institutional and interpersonal levels.^22^ The most critical challenges at the institutional level include lack of awareness of disease, access to healthcare, affordability, accountability and number of institutions and human resources.^24^ Context-specific programs may be required to foster and maintain a trusted interpersonal relationship between patients and healthcare providers.^22^ Initiatives undertaken by the Government involving lay health workers, to deliver health promotion activities for chronic diseases at the doorstep, may improve trust and promote task-sharing responsibilities to improve health utilisation for all, particularly women.^25^

Health insurance was associated with greater healthcare utilisation in public facilities. People with insurance were 28% more likely to utilise public healthcare, consistent with previous findings in LMICs.^26,27^ This observation should be interpreted with caution since insurance does not tend to cover outpatient services, which require out-of-pocket expenditure. However, people with government health insurance can usually access free inpatient healthcare from public facilities and some private hospitals that can claim fees from the government (so-called impanelled private hospitals) for managing chronic diseases.^28^ It is noteworthy that the number of impanelled private hospitals has increased in recent years thereby extending insurance coverage and financial protection for people with chronic diseases.

### Implications for future research

Our findings suggest that barriers to seeking public healthcare also exist at the level of the health systems, which are state-based and diverse in India. For example, people in the Rishi Valley more often reported difficulty in accessing healthcare (43.2%) than those residing in West Godavari (29.1%) or Trivandrum (8.8%). In addition, participants from Trivandrum in Kerala were much more likely to access public healthcare than those in the two sites in Andhra Pradesh. Arguably, Kerala has the best public healthcare system in India,^29^ and their high level of literacy likely contributes to better awareness of their health status.^29^ A positive impact of literacy and health awareness on healthcare utilisation is supported by the previous finding that individuals residing in regions of higher SEP were more likely to attend screening programs and utilise healthcare services than those in regions of lower SEP.^30,31^

SEP appears to affect the choice between public and private healthcare. Utilisation of public healthcare was twofold greater among people living below the poverty line than those who were not. However, these patterns of association were more marked in Kerala and West Godavari than in the Rishi Valley, suggesting that factors other than poverty also contribute. The perception of poor quality of public healthcare may also influence people with CMD to choose private facilities that usually provide higher quality care.^32^ Consistent with this, we found more than twice as many people who had greater household income (Quartile 2 to 4) sought care from private providers than people within the first quartile of household income. Finally, the factors that we identify herein, such as health insurance, type of ration card and household income merit deeper investigation to gain a greater understanding of their association with healthcare-seeking behaviour.

### Strengths and limitations

The current study is novel in that it provides reliable estimates of the overall prevalence of CMD and its risk factors in rural India, and provides these data disaggregated by sex. However, there are also some important limitations. First, self-reported prevalence surveys can be subject to the confounding effects of recall bias. However, recall bias was reduced by limiting inquiries on health-seeking to the last month. Second, limited health literacy or lack of screening among people with CMD may have led to under-reporting of CMD. In addition, the study represents just three regions in just two states, which may not reflect other regions as there are substantial disparities based on gender, SEP, health care institutions and geography between states.^33^ Despite these limitations, our findings were derived from a large sample from three diverse regions of India, so may provide generalisable insights into the barriers to, and potential enablers of, improved healthcare utilisation among rural residents with CMD.

## Conclusions

The prevalence of CMD is high in three diverse regions of rural India and is positively associated with indices of SEP even in rural populations. Furthermore, utilisation of health services, particularly public services, among those with CMD, is low. Major barriers to the utilisation of public health services include lack of health insurance and difficulty in accessing public healthcare.

## Supplemental Material

sj-docx-1-chi-10.1177_17423953231153550 - Supplemental material for Morbidity and utilisation of healthcare services among people with cardiometabolic disease in three diverse regions of rural India Click here for additional data file. Supplemental material, sj-docx-1-chi-10.1177_17423953231153550 for Morbidity and utilisation of healthcare services among people with cardiometabolic disease in three diverse regions of rural India by Sojib Bin Zaman, Roger G Evans, Clara K Chow, Rohina Joshi, Kavumpurathu R Thankappan, Brian Oldenburg, Ajay S Mahal, Kartik Kalyanram, Kamakshi Kartik, Michaela A Riddell, Oduru Suresh, Nihal Thomas, Gomathyamma K Mini, Pallab K Maulik, Velandai K Srikanth and Amanda G Thrift in Chronic Illness
