# Supplementary material for: Morbidity and utilisation of healthcare services among people with cardiometabolic disease in three diverse regions of rural India
Source: Chronic Illn. 2023 Feb 6;19(4):873–88. doi: 10.1177/17423953231153550 (PMC10655594; doi:10.1177/17423953231153550)
Supplement: sj-docx-1-chi-10.1177_17423953231153550 - Supplemental material for Morbidity and utilisation of healthcare services among people with cardiometabolic disease in three diverse regions of rural India [file sj-docx-1-chi-10.1177_17423953231153550.docx]

**Supplementary Materials**

**Morbidity and utilisation of healthcare services among people with cardiometabolic disease in three diverse regions of rural India**

Sojib Bin Zaman, Roger G. Evans, Clara K. Chow, Rohina Joshi, Kavumpurathu R. Thankappan, Brian Oldenburg, Ajay S. Mahal, Kartik Kalyanram, Kamakshi Kartik, Michaela A. Riddell, Oduru Suresh, Nihal Thomas, Gomathyamma K. Mini, Pallab K. Maulik, Velandai K. Srikanth, Amanda G. Thrift

**Table S1: STROBE Statement—Checklist of items included in the report**

|  | Item No | Recommendation | Page No |
| --- | --- | --- | --- |
| **Title and abstract** | 1 | (*a*) Indicate the study’s design with a commonly used term in the title or the abstract | 1 |
|  |  | (*b*) Provide in the abstract an informative and balanced summary of what was done and what was found | 2 |
| Introduction | | | |
| Background/rationale | 2 | Explain the scientific background and rationale for the investigation being reported | 3 |
| Objectives | 3 | State specific objectives, including any pre-specified hypotheses | 3 |
| Methods | | | |
| Study design | 4 | Present key elements of study design early in the paper | 4 |
| Setting | 5 | Describe the setting, locations, and relevant dates, including periods of recruitment, exposure, follow-up, and data collection | 4 |
| Participants | 6 | (*a*) Give the eligibility criteria, and the sources and methods of selection of participants | 4 |
| Variables | 7 | Clearly define all outcomes, exposures, predictors, potential confounders, and effect modifiers. Give diagnostic criteria, if applicable | Table S1 |
| Data sources/ measurement | 8 | For each variable of interest, give sources of data and details of methods of assessment (measurement). Describe comparability of assessment methods if there is more than one group | 4 |
| Bias | 9 | Describe any efforts to address potential sources of bias | 4 |
| Study size | 10 | Explain how the study size was arrived at | 4 |
| Quantitative variables | 11 | Explain how quantitative variables were handled in the analyses. If applicable, describe which groupings were chosen and why | 4 |
| Statistical methods | 12 | (*a*) Describe all statistical methods, including those used to control for confounding | 5 |
|  |  | (*b*) Describe any methods used to examine subgroups and interactions | 5 |
|  |  | (*c*) Explain how missing data were addressed | 5 |
|  |  | (*d*) If applicable, describe analytical methods taking account of sampling strategy | NA |
|  |  | (*e*) Describe any sensitivity analyses | NA |
| Results | | | |
| Participants | 13 | (a) Report numbers of individuals at each stage of study—eg numbers potentially eligible, examined for eligibility, confirmed eligible, included in the study, completing follow-up, and analysed | 6 |
|  |  | (b) Give reasons for non-participation at each stage | 6 |
|  |  | (c) Consider use of a flow diagram | NA (ref given) |
| Descriptive data | 14 | (a) Give characteristics of study participants (eg demographic, clinical, social) and information on exposures and potential confounders | Table 1 |
|  |  | (b) Indicate number of participants with missing data for each variable of interest | Tables 1-3 |
| Outcome data | 15 | Report numbers of outcome events or summary measures | 7-8 |
| Main results | 16 | (*a*) Give unadjusted estimates and, if applicable, confounder-adjusted estimates and their precision (eg, 95% confidence interval). Make clear which confounders were adjusted for and why they were included | Tables 1-3 |
|  |  | (*b*) Report category boundaries when continuous variables were categorised | Tables 1-3 |
|  |  | (*c*) If relevant, consider translating estimates of relative risk into absolute risk for a meaningful time period | NA |
| Other analyses | 17 | Report other analyses done—eg analyses of subgroups and interactions, and sensitivity analyses | Tables 1-3 |
| Discussion | | | |
| Key results | 18 | Summarise key results with reference to study objectives | 9 |
| Limitations | 19 | Discuss limitations of the study, taking into account sources of potential bias or imprecision. Discuss both direction and magnitude of any potential bias | 12 |
| Interpretation | 20 | Give a cautious overall interpretation of results considering objectives, limitations, multiplicity of analyses, results from similar studies, and other relevant evidence | 9-11 |
| Generalisability | 21 | Discuss the generalisability (external validity) of the study results | 12 |
| Other information | | | |
| Funding | 22 | Give the source of funding and the role of the funders for the present study and, if applicable, for the original study on which the present article is based | 12 |

NA: Not applicable; STROBE: Strengthening the Reporting of Observational Studies in Epidemiology ^1^

**Table S2. Health services and insurance in the study area, and data collection methods**

| **Healthcare service delivery and insurance system in the study area** |
| --- |
| This study, named “Control of Hypertension in Rural India” (CHIRI), was conducted from 2014 to 2016 in three rural regions of southern India. Of the three regions, Kerala is the most socioeconomically advantaged, the Rishi Valley region is the least, and the West Godavari District stands between these two extremes. Provision of public healthcare services differs across the study sites. For example, Kerala has intrinsically more robust primary, secondary, and tertiary medical care services and health status outcome compared to other states in India.^2, 3^ Additionally, Andhra Pradesh has a unique government-sponsored health insurance scheme where approximately 75% of the population is covered, whereas only approximately 30% have such coverage in Kerala.^4^ It is also relevant to consider that contextual changes occurred in India during and after the period of data collection for the current study (2014-2016). For example, Andhra Pradesh was divided into two states. Furthermore, a new scheme ‘Comprehensive Health Insurance Scheme (CHIS)’ was introduced in Kerala and Rashtriya Swasthya Bima Yojana (RSBY) scheme was launched nationally in 2008.^5^ The RSBY and CHIS were introduced to protect people living below poverty line (BPL) from major health shocks. In 2018, Pradhan Mantri Jan Arogya Yojana (PM-JAY) scheme was introduced nationally that subsumed the then RSBY scheme.^6^ |
| Data collection procedure |
| Data collectors underwent a 5-day training program on interview techniques and skills in the measurement of blood pressure (BP) and anthropometry according to the WHO STEP-wise approach to disease surveillance (WHO-STEPS) protocol.^7^ Questionnaires were used to obtain information about demographics, lifestyle, healthcare utilisation (public and private), healthcare-seeking behaviour, the types of healthcare facilities (e.g., primary health center/mobile clinic) they attended, and awareness of CMD. Hard copies of questionnaires were scanned at each region, sent to the coordinating center, captured and verified using Teleform Elite Version 9 software (Cardiff, San Jose, CA, USA), and uploaded to a Microsoft Access database. |

**Table S3. Classification and definition of variables**

| **Variable** | **Definition/ questions and cut-offs** | **Coding** |
| --- | --- | --- |
| Age | <50 years  ≥50 years | 0  1 |
| Sex | Men  Women | 0  1 |
| Marital status | Never married  Married  Separated/ divorced/ widow | 0  1  2 |
| Educational attainment | No formal schooling  Class 6 completed  7 -10 completed  ≥ Class 12 completed | 0  1  2  3 |
| Household income | Rs. 0 to 1000 (Q1)  Rs. >1000 to 1900 (Q2)  Rs. >1900 to 3000 (Q3)  Rs. >3000 (Q4) | 0  1  2  3 |
| Type of ration card | No ration / APL  Below Poverty Line  Poorest of the poor | 0  1  2 |
| Current smoker | Do you currently smoke cigarettes (filtered manufactured)/ hand rolled tobacco/ bidis (or local alternative e.g., cheroots, gurkha)? | 0=No  1= Yes |
| Ever taken alcohol | Have you ever consumed a drink that contains alcohol such as Indian made foreign liquor/spirits, country liquor, home grown liquor? | 0=No  1= Yes |
| Self-reported cardiometabolic disease (CMD)  [heart disease, stroke, chronic kidney disease, diabetes, hypertension, or hypercholesterolaemia] | We considered a participant to have cardiometabolic disease if they responded “yes” to the following questions:   - Have you ever been told by a doctor or other health worker that you have heart problems (including heart attack, coronary bypass surgery, and coronary angioplasty)? - Have you ever been told by a doctor or other health worker that you have symptoms suggestive of a stroke? (eg. weakness on one side of the body, visual disturbance, difficulty speaking or being understood) - Have you ever been told by a doctor or other health worker that you have chronic kidney disease? - Have you ever been told by a doctor or other health worker that you have diabetes (a high blood sugar)? - Have you ever been told by a doctor or other health worker that you had high blood pressure/ hypertension? - Have you ever been told by a doctor or other health worker that you had high blood fat/cholesterol? | 0=No  1= Yes |
| Healthcare sought in the last 3 months | Have you sought medical treatment or advice from anyone in the last 12 weeks (3 months)? | 0=No  1= Yes |
| Healthcare utilisation (public or private) | If yes, how many times did you seek treatment/advice in the past 4 weeks (in the last month)? | 0=None  1= One or more |
| Type of healthcare usually sought | From whom do you usually seek your health care? | 0= Private  1= Public |
| Health insurance | Do you have any kind of health care coverage such as health insurance? | 0=No  1= Yes |
| Literate | Ability to read and write | 0=No  1= Yes |
| Self-reported difficulty in getting to healthcare | When you need to see a doctor about your health, how easy/difficult is it for you to get there? | 0=Easy  1= Difficult |
| Physical activity | Are you physically active for more than 30 minutes 5 times a week or vigorously active 3 times per week? This includes physical activity during work, leisure or regular daily routine. | 0=No  1= Yes |
| Family history of CMD | Have any of your close relatives (mother, father, brothers, sisters, grandparents) been told they had high blood sugar (diabetes), or hypertension, or heart disease, or stroke? | 0=No  1= Yes |
| Screened for blood pressure (hypertension) | Have you ever had your blood pressure checked? | 0=No  1= Yes |
| Hypertension on blood pressure measurements | We considered a participant to have hypertension if their systolic blood pressure was ≥140 mmHg or their diastolic blood pressure was ≥90 mmHg or taking the blood pressure-lowering medication. | 0= No  1= Yes |
| Waist circumference (WC) above normal | WC, above normal, was considered for  Men waist >90 cm and Women waist >80 cm | 0= Normal  1= Above normal |

APL, above poverty line

**Table S4: Prevalence of hypertension, based on clinical measurements and self-report, stratified by gender and region**

| **Characteristics** | ***Stratified by Gender*** | | |  | **All sites combined (11657)** |  | ***Stratified by Region*** | | |  | |
| --- | --- | --- | --- | --- | --- | --- | --- | --- | --- | --- | --- |
|  | **Men (n=5784)** | **Women (n=5852)** | ***P _Gender_*** |  |  |  | **Rishi Valley (3400)** | **West Godavari (4500)** | **Trivandrum (3757)** | ***p _Region_*** | |
| **Hypertension** |  |  |  |  |  |  |  |  |  |  | |
| Clinical measurement | 1650 (28.5) | 1,810 (30.9) | **<0.001** |  | 3466 (29.7) |  | 779 (22.9) | 1383 (30.8) | 1304 (34.8) | **<0.001** | |
| Self-report | 990 (17.2) | 1505 (25.8) | **0.005** |  | 2501 (21.6) |  | 401 (11.9) | 1058 (23.5) | 1042 (27.7) | **<0.001** | |
| Data are presented as proportion (%) | | | | | | | | | | |  |
| P-values for comparing women to men and among three regions were generated using *X*^2^ tests | | | | | | | | | | |  |
| There were 21 missing observations on gender, so these are not included in the analysis of gender but they are included in the analysis of regions.  Clinical measurement of hypertension was defined according to measurement of BP (systolic blood pressure ≥140 mmHg and/or diastolic blood pressure ≥90 mmHg) and self-reported use of antihypertensive medications. | | | | | | | | | | |  |

**Table S5. Factors associated with self-reported cardiometabolic diseases among participants, all sites, multivariable logistic regression**

| **Characteristics** | **CMDs (10642)** | **Univariable analysis** | **Model 1** | **Model 2** | **Model 3** | **Model 4** | **Model 5** | **Model 6** |
| --- | --- | --- | --- | --- | --- | --- | --- | --- |
|  | **Yes (3629)** | **OR (95% CI)** | AOR (95% CI) | AOR (95% CI) | AOR (95% CI) | AOR (95% CI) | AOR (95% CI) | AOR (95% CI) |
| *Demographic* |  |  |  |  |  |  |  |  |
| **Age** |  |  |  |  |  |  |  |  |
| <50 years | 1159 (31.9) | Ref | Ref | Ref | Ref | Ref | Ref | Ref |
| ≥50 years | 2470 (68.1) | 5.02 (4.61, 5.48) | 5.08 (4.62, 5.59) | 4.65 (4.18, 5.17) | 4.57 (4.11, 5.08) | 5.08 (4.61, 5.59) | 4.58 (4.12, 5.09) | 4.65 (4.18, 5.16) |
| **Gender**^†^ |  |  |  |  |  |  |  |  |
| Men | 1612 (44.5) | Ref | Ref | Ref | Ref | Ref | Ref | Ref |
| Women | 2011 (55.5) | 1.38 (1.27, 1.49) | 1.29 (1.17, 1.41) | 1.27 (1.15, 1.4) | 1.26 (1.14, 1.39) | 1.28 (1.17, 1.41) | 1.26 (1.14, 1.39) | 1.26 (1.15, 1.39) |
| **Region** |  |  |  |  |  |  |  |  |
| Rishi Valley | 649 (17.8) | Ref | Ref | Ref | Ref | Ref | Ref |  |
| West Godavari | 1443 (39.7) | 1.56 (1.4, 1.74) | 1.8 (1.55, 2.08) | 1.77 (1.52, 2.05) | 1.76 (1.54, 2.02) | 1.8 (1.55, 2.08) | 1.76 (1.52, 2.05) | 1.77 (1.55, 2.03) |
| Trivandrum | 1537 (42.3) | 2.31 (2.06, 2.57) | 2.47 (2.13, 2.86) | 2.64 (2.26, 3.09) | 2.33 (2, 2.72) | 2.33 (1.98, 2.74) | 2.44 (2.07, 2.88) | 2.55 (2.21, 2.94) |
| *Socio-economic position* |  |  |  |  |  |  |  |  |
| **Education**^‡^ |  |  |  |  |  |  |  |  |
| No formal schooling | 952 (26.7) | Ref | _ | Ref | Ref | _ | Ref | Ref |
| Class 6 completed | 1059 (29.7) | 1.05 (0.94, 1.17) | _ | 1.12 (0.98, 1.27) | 1.11 (0.97, 1.26) | _ | 1.10 (0.97, 1.26) | 1.12 (0.96, 1.17) |
| 7 -11 completed | 1126 (31.6) | 0.84 (0.75, 0.93) | _ | 1.04 (0.90, 1.2) | 1.02 (0.88, 1.18) | _ | 1.01 (0.87, 1.16) | 1.05 (0.91, 1.22) |
| ≥Class 12 completed | 429 (12.0) | 0.48 (0.42, 0.54) | _ | 0.66 (0.55, 0.79) | 0.64 (0.54, 0.77) | _ | 0.63 (0.52, 0.75) | 0.69 (0.57, 0.82) |
| **Household income**^$^ |  |  |  |  |  |  |  |  |
| Rs 0 to 1000 (Q1) | 881 (24.3) | Ref | Ref | Ref | _ | Ref | Ref | - |
| >1000 to 1900 (Q2) | 634 (17.5) | 1.00 (0.89, 1.41) | 1.00 (0.86, 1.16) | 1.02 (0.88, 1.18) | _ | 1.00 (0.86, 1.16) | 1.02 (0.87, 1.18) | - |
| >1900 to 3000 (Q3) | 740 (20.4) | 0.97 (0.86, 1.09) | 0.94 (0.81, 1.09) | 0.95 (0.82, 1.11) | _ | 0.93 (0.8, 1.08) | 0.94 (0.81, 1.10) | - |
| Rs >3000 (Q4) | 744 (20.5) | 1.20 (1.11, 1.42) | 1.09 (0.93, 1.26) | 1.15 (0.99, 1.35) | _ | 1.06 (0.91, 1.24) | 1.13 (0.96, 1.31) | - |
| Missing group (Q0) | 630 (17.3) | 1.51 (1.35-1.78) | 0.9 (0.76, 1.07) | 0.92 (0.78, 1.09) | _ | 0.89 (0.76, 1.06) | 0.91 (0.77, 1.07) | - |
| **Ration type**^†^ |  |  |  |  |  |  |  |  |
| No ration card/ APL | 785 (22.6) | Ref | _ | _ | Ref | Ref | Ref | - |
| BPL | 2695 (77.4) | 0.58 (0.53, 0.63) | _ | _ | 0.82 (0.72, 0.93) | 0.9 (0.79, 1.02) | 0.83 (0.73, 0.95) | - |
| *Risk factors* |  |  |  |  |  |  |  |  |
| **Physical activity^α^**^§^ |  |  |  |  |  |  |  |  |
| No | 785 (22.6) | Ref | Ref | Ref | Ref | Ref | Ref | Ref |
| Yes | 2695 (77.4) | 0.65 (0.59, 0.72) | 0.78 (0.69, 0.89) | 0.75 (0.66, 0.85) | 0.76 (0.67, 0.86) | 0.79 (0.70, 0.89) | 0.76 (0.67, 0.86) | 0.75 (0.67, 0.82) |
| **Waist circumference**^β†^ |  |  |  |  |  |  |  |  |
| Normal | 1419 (39.4) | Ref | Ref | Ref | Ref | Ref | Ref | Ref |
| Above normal | 2188 (60.6) | 3.60 (3.30, 3.90) | 3.10 (2.81, 3.41) | 3.12 (2.83, 3.44) | 3.11 (2.82, 3.43) | 3.07 (2.78, 3.38) | 3.08 (2.79, 3.40) | 3.15 (2.86, 3.47) |

| Abbreviations: CI, confidence interval; OR, odds ratio; AOR, adjusted odds ratio; APL, above poverty line; BPL, below poverty line; CMD, cardiometabolic diseases  **^α^**Participants were considered as physically active if had performed exercises for more than 30 minutes 5 times a week or vigorously had worked out 3 times per week.  ^β^Waist circumference, above normal, was considered for men waist >90 cm and women waist >80 cm  Access to healthcare was classified as ‘easy’ and ‘difficult’ based on the perceptions and experiences of the interviewed participants |
| --- |
| Data are presented as odds ratios (95% confidence interval) |
| ^†^21-85 missing observations; ^‡^253 missing observations; ^§^843 missing observations. |
| There are also 1015 missing (don’t know) observations on CMD, and 843 missing observations on household income (hereafter called missing group, Q0), and these individuals were included in the multivariable logistic regression analysis. |
| Model 1 to 6: Each model includes adjustment for all other variables listed in that column  . |

**Table S6. Pattern of health service utilisation among people with cardiometabolic diseases, by gender**

| **Characteristics** | **Total** | **Men** | **Women** | ***P* _Gender_** |
| --- | --- | --- | --- | --- |
|  | **n = 3607** | **n = 1601** | **n = 2006** |  |
| **Health care sought** |  |  |  |  |
| In last 3 months^‡^ | 2142 (60.7) | 890 (57.1) | 1252 (63.6) | **<0.001** |
| In last month^†^ | 1498 (41.3) | 629 (39.0) | 869 (43.2) | **<0.001** |
| **Sought treatment from** |  |  |  |  |
| Private Hospital/Clinic^‡^ | 1470 (41.4) | 614 (38.9) | 856 (43.2) | **0.009** |
| Government/Public Hospital^‡^ | 609 (17.1) | 248 (15.6) | 361 (18.2) | **0.04** |
| Primary health centre^‡^ | 34 (0.9) | 18 (1.1) | 16 (0.8) | 0.31 |
| Mobile clinic^‡^ | 41 (1.1) | 22 (1.3) | 19 (0.9) | 0.23 |
| **From whom did you seek treatment** |  |  |  |  |
| Doctor/Physician^§^ | 613 (20.4) | 253 (19.2) | 360 (21.3) | 0.16 |
| Specialist doctor/Physician^§^ | 336 (11.2) | 164 (12.5) | 172 (10.2) | **0.04** |
| RMP^§^ | 180 (6.0) | 69 (5.2) | 111 (6.5) | 0.13 |
| Community Nurse^§^ | 12 (0.4) | 7 (0.5) | 5 (0.3) | 0.3 |
| ASHA^§^ | 7 (0.3) | 5 (0.3) | 2 (0.1) | 0.14 |
| **Health insurance**^†^ | 2493 (69.2) | 1102 (68.8) | 1391 (69.3) | 0.74 |
| Government insurance | 2397 (95.6) | 1062 (95.5) | 1335 (95.7) | 0.61 |
| Private insurance | 52 (1.4) | 22 (1.4) | 30 (1.5) | 0.74 |
| **Medical treatment payment** |  |  |  |  |
| Savings^‡^ | 1336 (37.4) | 559 (35.2) | 777 (39.1) | **0.01** |
| Government provided^‡^ | 411 (11.5) | 179 (11.2) | 232 (11.6) | 0.71 |
| Assistance/ gifts^‡^ | 131 (3.6) | 27 (1.7) | 104 (5.2) | **<0.001** |
| Unsecured loans^‡^ | 74 (2.1) | 50 (3.1) | 24 (1.2) | **<0.001** |
| **Healthcare accessibility**^†^ |  |  |  | **<0.001** |
| Easy | 2658 (73.4) | 1240 (77.0) | 1418 (70.5) |  |
| Difficult | 962 (26.6) | 370 (23.0) | 592 (29.5) |  |
| **Type of hospital admission**^‡^ |  |  |  |  |
| Private hospital | 282 (7.9) | 131 (8.3) | 151 (7.6) | 0.46 |
| Private Nursing home | 9 (0.2) | 3 (0.2) | 6 (0.3) | 0.5 |
| Private Medical College | 17 (0.4) | 9 (0.5) | 8 (0.4) | 0.47 |
| Govt./District/Tertiary Hospital | 130 (3.7) | 54 (3.4) | 76 (3.8) | 0.5 |
| **Medication:** |  |  |  |  |
| Self-reporting medication use† | 2580 (58.6) | 1140 (53.3) | 1440 (63.6) | 0.31 |
| Forget to take all medications^#^ |  |  |  | **0.001** |
| Never/very rarely | 2060 (82.7) | 928 (85.9) | 1132 (80.3) |  |
| Once in a while | 304 (12.2) | 108 (10.0) | 196 (13.9) |  |
| Sometimes/ usually/ all the time | 125 (5.0) | 44 (4.1) | 81 (5.7) |  |
| Abbreviations: RMP, Rural Medical Practitioner; ASHA, Accredited Social Health Activists | | | | |
| Data are presented as the proportion (%) | | | | |
| P-values for comparing women to men were generated using *X*^2^ tests | | | | |
| ^†^4-42 missing observations; ^‡^77-95 missing observations; ^§^625 missing observations. ^#^1,783 missing observations. There were also 6 missing observations on gender. These individuals were not included in the analysis. | | | | |
| Health insurance and medical treatment payments do not add up to 100%. | | | | |

**Table S7. Factors associated with healthcare utilisation in the previous month among people with self-reported cardiometabolic diseases, multivariable logistic regression**

| **Characteristics** | **Univariable analysis** | **Model 1** | **Model 2** | **Model 3** | **Model 4** | **Model 5** | **Model 6** |
| --- | --- | --- | --- | --- | --- | --- | --- |
|  | OR (95% CI) | AOR (95% CI) | AOR (95% CI) | AOR (95% CI) | AOR (95% CI) | AOR (95% CI) | AOR (95% CI) |
| *Demographic* |  |  |  |  |  |  |  |
| **Age group^†^** |  |  |  |  |  |  |  |
| <50 years | Ref |  |  | Ref | Ref | Ref | Ref |
| ≥50 years | 1.57 (1.36, 1.82) |  |  | 1.33 (1.12,1.57) | 1.33 (1.13, 1.58) | 1.43 (1.22, 1.68) | 1.34 (1.13, 1.58) |
| **Gender**^†^ |  |  |  |  |  |  |  |
| Men | Ref |  |  | Ref | Ref | Ref | Ref |
| Women | 1.19 (1.04, 1.36) |  |  | 1.16 (1.00, 1.34) | 1.14 (0.99, 1.32) | 1.2 (1.04, 1.39) | 1.16 (1, 1.34) |
| **Region** |  |  |  |  |  |  |  |
| Rishi Valley | Ref | Ref | ─ | Ref | Ref | Ref | Ref |
| West Godavari | 1.95 (1.60, 2.38) | 1.94 (1.59, 2.37) | ─ | 1.97 (1.53,2.53) | 1.98 (1.57, 2.50) | 2.01 (1.57, 2.58) | 1.97 (1.53, 2.53) |
| Trivandrum | 1.80 (1.48, 2.19) | 1.78 (1.46, 2.17) | ─ | 1.48 (1.12,1.95) | 1.52 (1.15, 2.00) | 1.46 (1.1, 1.94) | 1.52 (1.14, 2.03) |
| *Socio-economic position* |  |  |  |  |  |  |  |
| **Education** ^‡^ |  |  |  |  |  |  |  |
| No formal schooling | Ref | Ref | Ref | Ref | Ref | ─ | Ref |
| Class 6 completed | 1.02 (0.85, 1.21) | 1.08 (0.91, 1.30) | 0.98 (0.82, 1.18) | 0.96 (0.79, 1.17) | 0.96 (0.79, 1.16) | ─ | 0.96 (0.80, 1.17) |
| 7 -11 completed | 0.82 (0.69, 0.98) | 0.95 (0.79, 1.14) | 0.83 (0.68, 1.01) | 0.81 (0.66, 1.01) | 0.81 (0.66, 1.01) | ─ | 0.82 (0.66, 1.02) |
| ≥Class 12 completed | 0.58 (0.46, 0.74) | 0.72 (0.56, 0.93) | 0.63 (0.48, 0.82) | 0.72 (0.54, 0.96) | 0.71 (0.53, 0.95) | ─ | 0.73 (0.54, 0.98) |
| **Household income** ^$^ |  |  |  |  |  |  |  |
| Rs 0 to 1000 (Q1) | Ref | Ref | Ref | Ref | ─ | Ref | Ref |
| >1000 to 1900 (Q2) | 1.07 (0.87, 1.32) | 1.16 (0.94, 1.43) | 0.95 (0.77, 1.19) | 1.02 (0.81, 1.28) | ─ | 1.00 (0.80, 1.26) | 1.02 (0.81, 1.29) |
| >1900 to 3000 (Q3) | 1.24 (1.02, 1.52) | 1.31 (1.08, 1.61) | 1.06 (0.85, 1.31) | 1.12 (0.89, 1.41) | ─ | 1.1.0 (0.88, 1.39) | 1.12 (0.89, 1.41) |
| Rs >3000 (Q4) | 0.94 (0.77, 1.15) | 1.01 (0.83, 1.24) | 0.82 (0.66, 1.01) | 0.87 (0.69, 1.1) | ─ | 0.85 (0.67, 1.07) | 0.88 (0.70, 1.12) |
| Missing (Q0) |  | 1.21 (0.98, 1.49) | 0.96 (0.76, 1.2) | 0.95 (0.75, 1.2) | ─ | 0.95 (0.75, 1.2) | 0.95 (0.75, 1.21) |
| **Ration card**^†^ |  |  |  |  |  |  |  |
| No ration card/ APL | Ref | Ref | Ref | ─ | Ref | Ref | Ref |
| BPL | 1.07 (0.93, 1.23) | 1.09 (0.95, 1.26) | 1.31 (1.1, 1.55) | ─ | 1.10 (0.90, 1.33) | 1.12 (0.92, 1.36) | 1.07 (0.88, 1.30) |
| *Risk factors* |  |  |  |  |  |  |  |
| **CMD** |  |  |  |  |  |  |  |
| One | Ref | Ref | Ref | Ref | Ref | Ref | Ref |
| Two | 1.77 (1.51, 2.07) | 1.68 (1.43, 1.97) | 1.61 (1.37, 1.90) | 1.55 (1.31, 1.84) | 1.54 (1.30, 1.83) | 1.56 (1.31, 1.84) | 1.55 (1.31, 1.84) |
| ≥Three | 1.96 (1.63, 2.35) | 1.83 (1.51, 2.21) | 1.76 (1.45, 2.15) | 1.80 (1.47, 2.2) | 1.79 (1.46, 2.19) | 1.8 (1.47, 2.20) | 1.80 (1.47, 2.21) |
| **Family history of CMD** |  |  |  |  |  |  |  |
| No | Ref | Ref | Ref | Ref | Ref | Ref | Ref |
| Yes | 1.22 (1.06, 1.40) | 1.31 (1.14, 1.50) | 1.19 (1.03, 1.38) | 1.25 (1.07,1.46) | 1.25 (1.07,1.46) | 1.21 (1.04, 1.41) | 1.25 (1.07, 1.46) |
| *Health seeking behaviour* |  |  |  |  |  |  |  |
| **Health Insurance**^‡^ |  |  |  |  |  |  |  |
| No | Ref | Ref | Ref | Ref | Ref | Ref | Ref |
| Yes | 1.31 (1.14, 1.52) | 1.31 (1.13, 1.51) | 1.40 (1.20, 1.63) | 1.27 (1.08,1.49) | 1.26 (1.06,1.48) | 1.26 (1.07, 1.49) | 1.25 (1.06, 1.48) |
| **Screened for BP**^†^ |  |  |  |  |  |  |  |
| No | Ref | Ref | Ref | Ref | Ref | Ref | Ref |
| Yes | 2.74 (1.73, 4.37) | 2.40 (1.50, 3.83) | 2.06 (1.29, 3.31) | 2.30 (1.35, 3.9) | 2.30 (1.35, 3.91) | 2.39 (1.41, 4.06) | 2.30 (1.35, 3.91) |
| **Type of healthcare usually sought**^§^ |  |  |  |  |  |  |  |
| Private | Ref | Ref | Ref | Ref | Ref | Ref | Ref |
| Public | 1.41 (1.21, 1.63) | 1.43 (1.23, 1.66) | 1.60 (1.35, 1.91) | 1.57 (1.31,1.89) | 1.56 (1.30, 1.87) | 1.58 (1.31, 1.89) | 1.56 (1.30, 1.88) |
| Observation |  | ─ | ─ | 3,442 | 3,442 | 3,448 | 3,442 |
| AIC |  | ─ | ─ | 4500 | 4499 | 4509 | 4502 |
| BIC |  | ─ | ─ | 4611 | 4591 | 4608 | 4619 |

Abbreviations: CMD, cardiometabolic disease; APL, above poverty line; BPL, below poverty line; BP, blood pressure; CI, confidence interval; OR, odds ratio; AOR, adjusted odds ratio; AIC, Akaike's information criteria; BIC, Bayesian information criteria

Data are presented as odds ratios (95% confidence interval

^†^1-6 missing observations; ^‡^16-63 missing observations; ^§^150 missing observations; ^$^630 missing observations.

There are 4 missing observations on health care seeking, so these individuals were not included in the analysis.

There are also 1015 missing (don’t know) observations on cardiometabolic diseases. These individuals were not included in the analysis.

Model 1 to 6: Each model includes adjustment for all other variables listed in that column.

**Table S8: Factors associated with healthcare utilisation in the previous month among people with self-reported cardiometabolic diseases (n=647), Rishi Valley**

| **Characteristics** | **Univariable** | **Model 1** | **Model 2** | **Model 3** | **Model 4** | **Model 5** |
| --- | --- | --- | --- | --- | --- | --- |
|  | OR (95% CI) | AOR (95% CI) | AOR (95% CI) | AOR (95% CI) | AOR (95% CI) | AOR (95% CI) |
| **Age group** |  |  |  |  |  |  |
| <50 years | Ref | ─ | Ref | Ref | Ref | Ref |
| ≥50 years | 1.63 (1.11, 2.38) | ─ | 1.24 (0.77, 1.99) | 1.24 (0.77, 2.00) | 1.28 (0.81, 2.01) | 1.24 (0.77, 1.99) |
| **Gender** |  |  |  |  |  |  |
| Men | Ref | ─ | Ref | Ref | Ref | Ref |
| Women | 1.56 (1.11, 2.20) | ─ | 2.14 (1.35, 3.41) | 2.15 (1.34, 3.47) | 2.00 (1.29, 3.1) | 2.16 (1.34, 3.47) |
| **Education** |  |  |  |  |  |  |
| No formal schooling | Ref | Ref | Ref | Ref | ─ | Ref |
| Class 6 completed | 0.93 (0.61, 1.43) | 1.18 (0.74, 1.88) | 1.37 (0.81, 2.33) | 1.39 (0.82, 2.37) | ─ | 1.40 (0.82, 2.38) |
| 7 -11 completed | 0.57 (0.35, 0.95) | 0.85 (0.48, 1.48) | 0.85 (0.45, 1.61) | 0.85 (0.44, 1.62) | ─ | 0.85 (0.45, 1.63) |
| ≥Class 12 completed | 0.70 (0.36, 1.35) | 1.09 (0.53, 2.24) | 1.36 (0.59, 3.17) | 1.40 (0.59, 3.36) | ─ | 1.43 (0.59, 3.43) |
| **Household income** |  |  |  |  |  |  |
| Rs 0 to 1000 (Q1) | Ref | Ref | ─ | Ref | Ref | Ref |
| >1000 to 1900 (Q2) | 0.91 (0.53, 1.56) | 0.91 (0.53, 1.56) | ─ | 0.96 (0.52, 1.76) | 0.99 (0.54, 1.82) | 0.95 (0.52, 1.75) |
| >1900 to 3000 (Q3) | 1.20 (0.72, 2.01) | 1.2 (0.72, 2.01) | ─ | 1.35 (0.70, 2.58) | 1.35 (0.71, 2.56) | 1.35 (0.70, 2.59) |
| Rs >3000 (Q4) | 1.04 (0.61, 1.77) | 1.04 (0.61, 1.77) | ─ | 0.68 (0.30, 1.53) | 0.72 (0.32, 1.60) | 0.7 (0.31, 1.58) |
| Missing (Q0) | ─ | ─ | ─ | ─ | ─ | ─ |
| **Type of ration card** |  |  |  |  |  |  |
| No ration card/ APL | Ref | Ref | ─ | ─ | Ref | Ref |
| BPL | 1.43 (0.64, 3.21) | 1.35 (0.60, 3.06) | ─ | ─ | 1.14 (0.42, 3.12) | 1.21 (0.44, 3.34) |
| **CMD** |  |  |  |  |  |  |
| One | Ref | Ref | Ref | Ref | Ref | Ref |
| Two | 1.64 (1.05, 2.55) | 1.65 (1.06, 2.59) | 1.47 (0.85, 2.54) | 1.47 (0.85, 2.54) | 1.42 (0.83, 2.45) | 1.47 (0.85, 2.54) |
| ≥Three | 1.15 (0.55, 2.41) | 1.21 (0.57, 2.56) | 1.51 (0.65, 3.50) | 1.53 (0.66, 3.57) | 1.51 (0.66, 3.49) | 1.54 (0.66, 3.58) |
| **Family history of CMD** |  |  |  |  |  |  |
| No | Ref | Ref | Ref | Ref | Ref | Ref |
| Yes | 0.83 (0.55, 1.24) | 0.93 (0.61, 1.41) | 0.86 (0.51, 1.45) | 0.85 (0.5, 1.43) | 0.90 (0.55, 1.50) | 0.84 (0.50, 1.43) |
| **Health Insurance** |  |  |  |  |  |  |
| No | Ref | Ref | Ref | Ref | Ref | Ref |
| Yes | 1.09 (0.70, 1.7) | 1.18 (0.75, 1.85) | 1.24 (0.73, 2.09) | 1.20 (0.70, 2.03) | 1.15 (0.66, 1.98) | 1.16 (0.67, 2.01) |
| **Screened for BP** |  |  |  |  |  |  |
| No | Ref | Ref | Ref | Ref | Ref | Ref |
| Yes | 1.74 (0.85, 3.56) | 1.50 (0.73, 3.10) | 2.41 (0.91, 6.41) | 2.42 (0.91, 6.47) | 2.4 (0.90, 6.39) | 2.44 (0.91, 6.51) |
| **Type of healthcare usually sought** |  |  |  |  |  |  |
| Private | Ref | Ref | Ref | Ref | Ref | Ref |
| Public | 1.38 (0.84, 2.25) | 1.4 (0.85, 2.30) | 1.43 (0.86, 2.38) | 1.45 (0.87, 2.41) | 1.49 (0.90, 2.48) | 1.44 (0.87, 2.40) |
| Observation | ─ | ─ | 495 | 495 | 496 | 495 |
| AIC | ─ | ─ | 583 | 587 | 587 | 589 |
| BIC | ─ | ─ | 634 | 650 | 641 | 656 |

Abbreviations: CMD, cardiometabolic disease; APL, above poverty line; BPL, below poverty line; BP, blood pressure; CI, confidence interval; OR, odds ratio; AOR, adjusted odds ratio; AIC, Akaike's information criteria; BIC, Bayesian information criteria

Data are presented as odds ratios (95% confidence interval

Model 1 to 5: Each model includes adjustment for all other variables listed in that column.

**Table S9: Factors associated with healthcare utilisation in the previous month among people with self-reported cardiometabolic diseases (n=1443), West Godavari**

| **Characteristics** | **Univariable** | **Model 1** | **Model 2** | **Model 3** | **Model 4** | **Model 5** |
| --- | --- | --- | --- | --- | --- | --- |
|  | OR (95% CI) | AOR (95% CI) | AOR (95% CI) | AOR (95% CI) | AOR (95% CI) | AOR (95% CI) |
| **Age group** |  |  |  |  |  |  |
| <50 years | Ref | ─ | Ref | Ref | Ref | Ref |
| ≥50 years | 1.55 (1.24, 1.94) | ─ | 1.35 (1.05, 1.73) | 1.36 (1.05, 1.74) | 1.5 (1.17, 1.90) | 1.37 (1.07, 1.77) |
| **Gender** |  |  |  |  |  |  |
| Men | Ref | ─ | Ref | Ref | Ref | Ref |
| Women | 0.93 (0.76, 1.15) | ─ | 0.87 (0.69, 1.09) | 0.90 (0.71, 1.13) | 0.97 (0.78, 1.21) | 0.90 (0.71, 1.13) |
| **Education** |  |  |  |  |  |  |
| No formal schooling | Ref | Ref | Ref | Ref | ─ | Ref |
| Class 6 completed | 1.01 (0.79, 1.28) | 1.04 (0.81, 1.32) | 0.96 (0.74, 1.24) | 0.97 (0.75, 1.26) | ─ | 0.97 (0.75, 1.26) |
| 7 -11 completed | 0.78 (0.57, 1.05) | 0.86 (0.62, 1.18) | 0.73 (0.52, 1.03) | 0.74 (0.53, 1.05) | ─ | 0.75 (0.53, 1.07) |
| ≥Class 12 completed | 0.52 (0.33, 0.85) | 0.56 (0.34, 0.92) | 0.51 (0.30, 0.87) | 0.54 (0.32, 0.93) | ─ | 0.56 (0.33, 0.97) |
| **Household income** |  |  |  |  |  |  |
| Rs 0 to 1000 (Q1) | Ref | Ref | ─ | Ref | Ref | Ref |
| >1000 to 1900 (Q2) | 0.82 (0.57, 1.19) | 0.89 (0.61, 1.28) | ─ | 0.97 (0.66, 1.42) | 0.97 (0.66, 1.41) | 0.97 (0.66, 1.42) |
| >1900 to 3000 (Q3) | 0.92 (0.65, 1.29) | 0.96 (0.68, 1.36) | ─ | 1.02 (0.72, 1.46) | 1.02 (0.71, 1.46) | 1.03 (0.72, 1.47) |
| Rs >3000 (Q4) | 0.74 (0.52, 1.05) | 0.80 (0.56, 1.14) | ─ | 0.85 (0.59, 1.24) | 0.84 (0.58, 1.23) | 0.87 (0.60, 1.27) |
| Missing (Q0) | 0.63 (0.36, 1.10) | 0.64 (0.36, 1.13) | ─ | 0.71 (0.39, 1.29) | 0.70 (0.39, 1.27) | 0.73 (0.40, 1.32) |
| **Type of ration card** |  |  |  |  |  |  |
| No ration card/ APL | Ref | Ref | ─ | ─ | Ref | Ref |
| BPL | 1.37 (1.00, 1.88) | 1.43 (1.04, 1.96) | ─ | ─ | 1.30 (0.89, 1.91) | 1.21 (0.82, 1.78) |
| **CMD** |  |  |  |  |  |  |
| One | Ref | Ref | Ref | Ref | Ref | Ref |
| Two | 1.60 (1.26, 2.04) | 1.52 (1.19, 1.95) | 1.49 (1.16, 1.92) | 1.49 (1.16, 1.92) | 1.48 (1.15, 1.90) | 1.50 (1.16, 1.93) |
| ≥Three | 2.10 (1.49, 2.97) | 2.00 (1.41, 2.83) | 2.10 (1.47, 3.02) | 2.13 (1.49, 3.06) | 2.03 (1.42, 2.90) | 2.15 (1.50, 3.09) |
| **Family history of CMD** |  |  |  |  |  |  |
| No | Ref | Ref | Ref | Ref | Ref | Ref |
| Yes | 1.13 (0.92, 1.39) | 1.22 (0.99, 1.51) | 1.30 (1.03, 1.63) | 1.29 (1.03, 1.63) | 1.23 (0.99, 1.54) | 1.30 (1.03, 1.63) |
| **Health Insurance** |  |  |  |  |  |  |
| No | Ref | Ref | Ref | Ref | Ref | Ref |
| Yes | 1.29 (1.01, 1.66) | 1.28 (1.00, 1.65) | 1.26 (0.97, 1.64) | 1.24 (0.95, 1.61) | 1.17 (0.87, 1.57) | 1.16 (0.86, 1.56) |
| **Screened for BP** |  |  |  |  |  |  |
| No | Ref | Ref | Ref | Ref | Ref | Ref |
| Yes | 1.97 (0.86, 4.52) | 1.77 (0.77, 4.11) | 1.39 (0.59, 3.29) | 1.39 (0.59, 3.29) | 1.55 (0.66, 3.62) | 1.37 (0.58, 3.23) |
| **Type of healthcare usually sought** | |  |  |  |  |  |
| Private | Ref | Ref | Ref | Ref | Ref | Ref |
| Public | 1.33 (0.86, 2.07) | 1.31 (0.84, 2.03) | 1.33 (0.84, 2.10) | 1.32 (0.84, 2.08) | 1.31 (0.83, 2.05) | 1.31 (0.83, 2.06) |
| Observation | ─ | ─ | 1,416 | 1,416 | 1,421 | 1,416 |
| AIC | ─ | ─ | 1918 | 1923 | 1932 | 1924 |
| BIC | ─ | ─ | 1981 | 2007 | 2005 | 2014 |
| Abbreviations: CMD, cardiometabolic disease; APL, above poverty line; BPL, below poverty line; BP, blood pressure; CI, confidence interval; OR, odds ratio; AOR, adjusted odds ratio; AIC, Akaike's information criteria; BIC, Bayesian information criteria | | | | | | |
| Data are presented as odds ratios (95% confidence interval  Model 1 to 5: Each model includes adjustment for all other variables listed in that column. | | | | | | |

**Table S10: Factors associated with healthcare utilisation in the previous month among people with self-reported cardiometabolic diseases (n=1,535), Trivandrum**

| **Characteristics** | **Univariable** | | **Model 1** | | **Model 2** | | **Model 3** | | **Model 4** | | **Model 5** | |
| --- | --- | --- | --- | --- | --- | --- | --- | --- | --- | --- | --- | --- |
|  | OR (95% CI) | | AOR (95% CI) | | AOR (95% CI) | | AOR (95% CI) | | AOR (95% CI) | | AOR (95% CI) | |
| **Age group** |  | |  | |  | |  | |  | |  | |
| <50 years | Ref | | ─ | | Ref | | Ref | | Ref | | Ref | |
| ≥50 years | 1.59 (1.27, 1.98) | | ─ | | 1.32 (1.02, 1.71) | | 1.34 (1.03, 1.73) | | 1.43 (1.12, 1.83) | | 1.34 (1.03, 1.75) | |
| **Gender** |  | |  | |  | |  | |  | |  | |
| Men | Ref | | ─ | | Ref | | Ref | | Ref | | Ref | |
| Women | 1.26 (1.03, 1.55) | | ─ | | 1.23 (0.99, 1.54) | | 1.24 (0.99, 1.56) | | 1.27 (1.02, 1.58) | | 1.24 (1.00, 1.56) | |
| **Education** |  | |  | |  | |  | |  | |  | |
| No formal schooling | Ref | | Ref | | Ref | | Ref | | ─ | | Ref | |
| Class 6 completed | 0.81 (0.57, 1.14) | | 0.87 (0.61, 1.23) | | 0.89 (0.62, 1.29) | | 0.90 (0.62, 1.29) | | ─ | | 0.90 (0.62, 1.29) | |
| 7 -11 completed | 0.65 (0.48, 0.88) | | 0.77 (0.56, 1.05) | | 0.83 (0.60, 1.16) | | 0.84 (0.60, 1.16) | | ─ | | 0.84 (0.60, 1.18) | |
| ≥Class 12 completed | 0.44 (0.31, 0.64) | | 0.56 (0.38, 0.82) | | 0.71 (0.47, 1.07) | | 0.73 (0.48, 1.10) | | ─ | | 0.74 (0.48, 1.13) | |
| **Household income** |  | |  | |  | |  | |  | |  | |
| Rs 0 to 1000 (Q1) | Ref | | Ref | | ─ | | Ref | | Ref | | Ref | |
| >1000 to 1900 (Q2) | 0.97 (0.69, 1.37) | | 1.04 (0.74, 1.48) | | ─ | | 1.12 (0.78, 1.60) | | 1.10 (0.77, 1.58) | | 1.12 (0.78, 1.60) | |
| >1900 to 3000 (Q3) | 1.04 (0.72, 1.51) | | 1.14 (0.78, 1.65) | | ─ | | 1.28 (0.87, 1.88) | | 1.25 (0.85, 1.84) | | 1.28 (0.87, 1.88) | |
| Rs >3000 (Q4) | 0.71 (0.51, 1.00) | | 0.76 (0.54, 1.07) | | ─ | | 0.94 (0.66, 1.35) | | 0.91 (0.63, 1.30) | | 0.95 (0.66, 1.37) | |
| Missing (Q0) | 1.05 (0.80, 1.39) | | 1.03 (0.78, 1.36) | | ─ | | 1.06 (0.79, 1.41) | | 1.05 (0.79, 1.40) | | 1.06 (0.79, 1.41) | |
| **Type of ration card** |  | |  | |  | |  | |  | |  | |
| No ration card/ APL | Ref | | Ref | | ─ | | ─ | | Ref | | Ref | |
| BPL | 1.23 (1.00, 1.52) | | 1.27 (1.02, 1.57) | | ─ | | ─ | | 1.08 (0.85, 1.36) | | 1.04 (0.81, 1.32) | |
| **CMD** |  | |  | |  | |  | |  | |  | |
| One | Ref | | Ref | | Ref | | Ref | | Ref | | Ref | |
| Two | 1.84 (1.44, 2.34) | | 1.72 (1.34, 2.2) | | 1.66 (1.29, 2.15) | | 1.67 (1.29, 2.16) | | 1.70 (1.31, 2.19) | | 1.67 (1.29, 2.16) | |
| ≥Three | 1.95 (1.52, 2.5) | | 1.80 (1.38, 2.33) | | 1.78 (1.36, 2.33) | | 1.78 (1.36, 2.34) | | 1.82 (1.39, 2.38) | | 1.79 (1.36, 2.34) | |
| **Family history of CMD** |  | |  | |  | |  | |  | |  | |
| No | Ref | | Ref | | Ref | | Ref | | Ref | | Ref | |
| Yes | 1.15 (0.93, 1.43) | | 1.25 (1.00, 1.57) | | 1.30 (1.03, 1.64) | | 1.29 (1.02, 1.64) | | 1.27 (1.01, 1.6) | | 1.30 (1.03, 1.64) | |
| **Health Insurance** |  | |  | |  | |  | |  | |  | |
| No | Ref | | Ref | | Ref | | Ref | | Ref | | Ref | |
| Yes | 1.57 (1.28, 1.93) | | 1.56 (1.26, 1.92) | | 1.32 (1.06, 1.65) | | 1.31 (1.05, 1.64) | | 1.32 (1.05, 1.65) | | 1.3 (1.04, 1.63) | |
| **Screened for BP** |  | |  | |  | |  | |  | |  | |
| No | Ref | | Ref | | Ref | | Ref | | Ref | | Ref | |
| Yes | 4.33 (1.66, 11.29) | | 3.56 (1.36, 9.33) | | 3.41 (1.28, 9.07) | | 3.43 (1.29, 9.12) | | 3.49 (1.31, 9.26) | | 3.44 (1.29, 9.16) | |
| **Type of healthcare usually sought** | |  | |  | |  | |  | |  | |  |
| Private | Ref | | Ref | | Ref | | Ref | | Ref | | Ref | |
| Public | 1.66 (1.35, 2.03) | | 1.73 (1.41, 2.13) | | 1.67 (1.34, 2.08) | | 1.67 (1.34, 2.09) | | 1.68 (1.34, 2.11) | | 1.66 (1.33, 2.09) | |
| Observation | ─ | | ─ | | 1,531 | | 1,531 | | 1,531 | | 1,531 | |
| AIC | ─ | | ─ | | 2015 | | 2020 | | 2018 | | 2022 | |
| BIC | ─ | | ─ | | 2079 | | 2105 | | 2093 | | 2113 | |
| Abbreviations: CMD, cardiometabolic disease; APL, above poverty line; BPL, below poverty line; BP, blood pressure; CI, confidence interval; OR, odds ratio; AOR, adjusted odds ratio; AIC, Akaike's information criteria; BIC, Bayesian information criteria | | | | | | | | | | | | |
| Data are presented as the proportion (%) or odds ratios (95% confidence interval)  Model 1 to 5: Each model includes adjustment for all other variables listed in that column. | | | | | | | | | | | | |

**Table S11: Association of current medication use with healthcare utilisation in the previous month among people with self-reported cardiometabolic diseases, stratified by educational attainment**

| **Schooling** | **Medication use** |  | **Healthcare utilisation** | |  | **Univariable** |  | **Multivariable** |  |
| --- | --- | --- | --- | --- | --- | --- | --- | --- | --- |
|  |  |  | No | Yes |  | OR (95% CI) |  | AOR (95% CI) |  |
| Overall | No medication use |  | 928 (43.67) | 253 (16.88) |  | Ref |  | Ref |  |
|  | Self-reported medication use |  | 1197 (56.33) | 1246 (83.12) |  | 3.82 (3.25, 4.48) |  | 4.05 (1.26, 13.08) |  |
| Stratified by Schooling | |  |  |  |  |  |  |  |  |
| No schooling | |  |  |  |  |  |  |  |  |
|  | No medication use |  | 229 (43.37) | 69 (16.35) |  | Ref |  | Ref |  |
|  | Self-reported medication use |  | 299 (56.63) | 353 (83.65) |  | 3.92 (2.87, 5.34) |  | 3.84 (2.81, 5.26) |  |
| Class 6 completed | |  |  |  |  |  |  |  |  |
|  | No medication use |  | 222 (38.01) | 55 (11.58) |  | Ref |  | Ref |  |
|  | Self-reported medication use |  | 362 (61.99) | 420 (88.42) |  | 4.68 (3.38, 6.49) |  | 4.47 (3.2, 6.23) |  |
| 7 -11 completed | |  |  |  |  |  |  |  |  |
|  | No medication use |  | 300 (44.12) | 84 (19.43) |  | Ref |  | Ref |  |
|  | Self-reported medication use |  | 380 (55.88) | 362 (81.17) |  | 3.40 (2.56, 4.5) |  | 3.60 (2.66, 4.87) |  |
| ≥Class 12 |  |  |  |  |  |  |  |  |  |
|  | No medication use |  | 151 (51.71) | 39 (28.68) |  | Ref |  | Ref |  |
|  | Self-reported medication use |  | 141 (48.29) | 97 (71.32) |  | 4.04 (1.28, 12.75) |  | 2.79 (1.73, 4.52) |  |
| Abbreviations: CI, confidence interval; OR, odds ratio; AOR, adjusted odds ratio | | | | | | | | | |
| Data are presented as the proportion (%) or odds ratios (95% confidence interval)  There are also 1015 missing (don’t know) observations on cardiometabolic diseases. These individuals were not included in the analysis. | | | | | | | | | |
| Multivariable logistic regression is adjusted for age and gender | | | | | | | | | |

**Table S12: Factors associated with utilisation of private healthcare providers among people with self-reported cardiometabolic diseases, all sites, multivariable logistic regression**

| **Characteristics** | **Univariable** | **Model 1** | **Model 2** | **Multivariable** |
| --- | --- | --- | --- | --- |
|  | OR (95% CI) | AOR (95% CI) | AOR (95% CI) | AOR (95% CI) |
| **Age group** |  |  |  |  |
| <50 years | Ref | ─ | ─ | Ref |
| ≥50 years | 1.09 (0.93, 1.28) | ─ | ─ | 1.24 (1.00, 1.54) |
| **Gender**^†^ |  |  |  |  |
| Men | Ref | ─ | ─ | Ref |
| Women | 1.02 (0.88, 1.19) | ─ | ─ | 1.33 (1.10, 1.60) |
| **Region** |  |  |  |  |
| Rishi Valley | Ref | Ref | ─ | Ref |
| West Godavari | 3.50 (2.55, 4.8) | 3.45 (2.51, 4.73) | ─ | 2.65 (1.87, 3.74) |
| Trivandrum | 0.19 (0.15, 0.25) | 0.19 (0.15, 0.25) | ─ | 0.06 (0.05, 0.09) |
| **Education**^‡^ |  |  |  |  |
| No schooling | Ref | Ref | Ref | Ref |
| Class 6 completed | 1.10 (0.89, 1.36) | 1.09 (0.88, 1.35) | 1.32 (1.02, 1.72) | 1.22 (0.94, 1.60) |
| 7 -11 completed | 0.55 (0.45, 0.67) | 0.54 (0.44, 0.66) | 1.99 (1.54, 2.59) | 1.43 (1.09, 1.88) |
| ≥Class 12 | 0.70 (0.54, 0.90) | 0.67 (0.51, 0.88) | 2.96 (2.13, 4.11) | 1.82 (1.28, 2.60) |
| **Household income**^§^ |  |  |  |  |
| Rs 0 to 1000 (Q1) | Ref | Ref | Ref | Ref |
| >1000 to 1900 (Q2) | 1.84 (1.45, 2.34) | 1.93 (1.52, 2.45) | 1.70 (1.28, 2.25) | 1.56 (1.16, 2.09) |
| >1900 to 3000 (Q3) | 2.33 (1.83, 2.95) | 2.41 (1.90, 3.07) | 1.53 (1.14, 2.04) | 1.41 (1.04, 1.91) |
| Rs >3000 (Q4) | 2.37 (1.86, 3.01) | 2.47 (1.94, 3.14) | 2.36 (1.78, 3.15) | 1.61 (1.19, 2.18) |
| Missing (Q0) | 0.54 (0.44, 0.67) | 0.53 (0.42, 0.65) | 1.31 (1.01, 1.68) | 1.13 (0.87, 1.48) |
| **Type of ration card**^†^ |  |  |  |  |
| No ration card/ APL | Ref | Ref | Ref | Ref |
| BPL | 1.82 (1.57, 2.12) | 1.83 (1.57, 2.13) | 0.33 (0.27, 0.41) | 0.46 (0.37, 0.59) |
| **CMD** |  |  |  |  |
| One | Ref | Ref | Ref | Ref |
| Two | 0.88 (0.74, 1.06) | 0.86 (0.72, 1.03) | 1.19 (0.96, 1.47) | 1.27 (1.02, 1.59) |
| ≥Three | 0.69 (0.57, 0.84) | 0.66 (0.54, 0.81) | 1.42 (1.12, 1.80) | 1.46 (1.14, 1.88) |

| **Health insurance**^†^ |  |  |  |  |
| --- | --- | --- | --- | --- |
| No | Ref | Ref | Ref | Ref |
| Yes | 0.78 (0.66, 0.92) | 0.78 (0.66, 0.92) | 0.35 (0.29, 0.43) | 0.43 (0.35, 0.52) |
| Observation | ─ | ─ | ─ | 3,450 |
| AIC | ─ | ─ | ─ | 3029.9 |
| BIC | ─ | ─ | ─ | 3128.3 |
| Abbreviations: APL, above poverty line; BPL, below poverty line; CI, confidence interval; OR, odds ratio; AOR, adjusted odds ratio; CMD, cardiometabolic diseases; AIC, Akaike's information criteria; BIC, Bayesian information criteria | | | | |
| Data are presented as odds ratios (95% confidence interval) | | | | |
| There are 630 missing observations on income and these are included in the multivariable logistic regression | | | | |
| ^†^2-16 missing observations; ^‡^63 missing observations; ^§^630 missing observations.  There are 150 missing observations on health care providers, so these individuals were not included in the analysis.  There are also 1015 missing (don’t know) observations on cardiometabolic diseases. These individuals were not included in the analysis.  Model 1 to 2: Each model includes adjustment for all other variables listed in that column.  Multivariable model: Adjusted for all variables listed in that column. | | | | |

**Table S13: Factors associated with utilisation of public healthcare providers among people with self-reported cardiometabolic diseases, all sites, multivariable logistic regression**

| **Characteristics** | **Model 1** | **Model 2** | **Model 3** | **Model 4** | **Model 5** | **Multivariable** |
| --- | --- | --- | --- | --- | --- | --- |
|  | AOR (95% CI) | AOR (95% CI) | AOR (95% CI) | AOR (95% CI) | AOR (95% CI) | AOR (95% CI) |
| **Age group** |  |  |  |  |  |  |
| <50 years | ─ | ─ | Ref | Ref | Ref | Ref |
| ≥50 years | ─ | ─ | 0.75 (0.61, 0.92) | 0.73 (0.60, 0.90) | 0.96 (0.79, 1.17) | 0.8 (0.65, 1.00) |
| **Gender**^†^ |  |  |  |  |  |  |
| Men | ─ | ─ | Ref | Ref | Ref | Ref |
| Women | ─ | ─ | 0.77 (0.64, 0.92) | 0.74 (0.62, 0.89) | 0.84 (0.7, 1) | 0.75 (0.62, 0.91) |
| **Region** |  |  |  |  |  |  |
| Rishi valley | Ref | ─ | Ref | Ref | Ref | Ref |
| West Godavari | 0.29 (0.21, 0.40) | ─ | 0.30 (0.22, 0.42) | 0.38 (0.27, 0.54) | 0.31 (0.23, 0.43) | 0.38 (0.27, 0.53) |
| Trivandrum | 5.22 (4.07, 6.71) | ─ | 10.32 (7.7, 13.84) | 11.23 (8.22, 15.36) | 13.27 (9.77, 18.02) | 15.66 (11.23, 21.84) |
| **Education**^‡^ |  |  |  |  |  |  |
| No schooling | Ref | Ref | Ref | Ref | ─ | Ref |
| Class 6 completed | 0.92 (0.74, 1.14) | 0.76 (0.58, 0.98) | 0.79 (0.6, 1.03) | 0.80 (0.61, 1.04) | ─ | 0.82 (0.63, 1.07) |
| 7 -11 completed | 1.86 (1.52, 2.28) | 0.5 (0.39, 0.65) | 0.57 (0.44, 0.74) | 0.61 (0.47, 0.79) | ─ | 0.70 (0.53, 0.92) |
| ≥Class 12 | 1.50 (1.14, 1.96) | 0.34 (0.24, 0.47) | 0.38 (0.27, 0.54) | 0.44 (0.31, 0.63) | ─ | 0.55 (0.38, 0.78) |
| **Household income**^§^ |  |  |  |  |  |  |
| Rs 0 to 1000 (Q1) | Ref | Ref | ─ | Ref | ─ | Ref |
| >1000 to 1900 (Q2) | 0.52 (0.41, 0.66) | 0.59 (0.44, 0.78) | ─ | 0.63 (0.47, 0.85) | ─ | 0.64 (0.48, 0.86) |
| >1900 to 3000 (Q3) | 0.41 (0.33, 0.53) | 0.65 (0.49, 0.87) | ─ | 0.69 (0.51, 0.93) | ─ | 0.71 (0.52, 0.96) |
| Rs >3000 (Q4) | 0.41 (0.32, 0.52) | 0.42 (0.32, 0.56) | ─ | 0.55 (0.41, 0.74) | ─ | 0.62 (0.46, 0.84) |
| Missing (Q0) | 1.90 (1.53, 2.35) | 0.77 (0.59, 0.99) | ─ | 0.82 (0.63, 1.07) | ─ | 0.88 (0.68, 1.15) |
| **Type of ration card†** |  |  |  |  |  |  |
| No ration card/ APL | Ref | Ref | ─ | ─ | Ref | Ref |
| BPL | 0.55 (0.47, 0.64) | 3.03 (2.44, 3.76) | ─ | ─ | 2.47 (1.97, 3.10) | 2.16 (1.71, 2.73) |
| **CMD** |  |  |  |  |  |  |
| One | Ref | Ref | Ref | Ref | Ref | Ref |
| Two | 1.16 (0.97, 1.39) | 0.84 (0.68, 1.04) | 0.78 (0.63, 0.97) | 0.78 (0.63, 0.97) | 0.81 (0.65, 1.01) | 0.79 (0.63, 0.98) |
| ≥Three | 1.52 (1.24, 1.86) | 0.70 (0.56, 0.89) | 0.65 (0.51, 0.83) | 0.66 (0.51, 0.85) | 0.71 (0.55, 0.91) | 0.68 (0.53, 0.88) |
| **Health insurance**^†^ |  |  |  |  |  |  |
| No | Ref | Ref | Ref | Ref | Ref | Ref |
| Yes | 1.28 (1.09, 1.50) | 2.83 (2.33, 3.44) | 2.67 (2.19, 3.25) | 2.65 (2.17, 3.23) | 2.42 (1.98, 2.96) | 2.35 (1.92, 2.88) |
| Observation | ─ | ─ | 3450 | 3450 | 3456 | 3450 |
| AIC | ─ | ─ | 3082 | 3071 | 3054 | 3030 |
| BIC | ─ | ─ | 3150 | 3163 | 3109 | 3128 |
| Abbreviations: APL, above poverty line; BPL, below poverty line; CI, confidence interval; OR, odds ratio; AOR, adjusted odds ratio; CMD, cardiometabolic disease; AIC, Akaike's information criteria; BIC, Bayesian information criteria | | | | | | |
| Data are presented as odds ratios (95% confidence interval) | | | | | | |
| There are 630 missing observations on income and these are included in the multivariable logistic regression | | | | | | |
| ^†^2-16 missing observations; ^‡^63 missing observations; ^§^630 missing observations.  There are 150 missing observations on health care providers, so these individuals were not included in the analysis.  There are also 1015 missing (don’t know) observations on cardiometabolic diseases. These individuals were not included in the analysis.  Model 1 to 5: Each model includes adjustment for all other variables listed in that column.  Multivariable model: Adjusted for all variables listed in that column. | | | | | | |

**Table S14: Factors associated with utilisation of public healthcare providers among people with self-reported cardiometabolic diseases, by regions, multivariable logistic regression**

|  | **Rishi Valley** | |  | **West Godavari** | |  | **Trivandrum** | |
| --- | --- | --- | --- | --- | --- | --- | --- | --- |
| **Characteristics** | **Univariable** | **Multivariable** |  | **Univariable** | **Multivariable** |  | **Univariable** | **Multivariable** |
|  | OR (95% CI) | AOR (95% CI) |  | OR (95% CI) | AOR (95% CI) |  | OR (95% CI) | AOR (95% CI) |
| **Age group** |  |  |  |  |  |  |  |  |
| <50 years | Ref | Ref |  | Ref | Ref |  | Ref | Ref |
| ≥50 years | 1.38 (0.83, 2.28) | 1.17 (0.68, 2.03) |  | 1.20 (0.74, 1.95) | 1.08 (0.63, 1.85) |  | 0.76 (0.61, 0.94) | 0.67 (0.51, 0.87) |
| **Gender**^†^ |  |  |  |  |  |  |  |  |
| Men | Ref | Ref |  | Ref | Ref |  | Ref | Ref |
| Women | 0.85 (0.54, 1.34) | 0.72 (0.43, 1.23) |  | 0.71 (0.46, 1.10) | 0.63 (0.40, 1.01) |  | 0.93 (0.76, 1.14) | 0.78 (0.62, 0.98) |
| **Education**^‡^ |  |  |  |  |  |  |  |  |
| No schooling | Ref | Ref |  | Ref | Ref |  | Ref | Ref |
| Class 6 completed | 1.18 (0.69, 2.02) | 1.09 (0.62, 1.94) |  | 0.67 (0.42, 1.08) | 0.70 (0.42, 1.15) |  | 0.79 (0.55, 1.13) | 0.80 (0.54, 1.17) |
| 7 -11 completed | 0.54 (0.27, 1.10) | 0.53 (0.25, 1.14) |  | 0.44 (0.21, 0.89) | 0.50 (0.23, 1.07) |  | 0.63 (0.46, 0.86) | 0.77 (0.54, 1.09) |
| ≥Class 12 | 0.82 (0.34, 1.97) | 0.88 (0.33, 2.36) |  | 0.25 (0.06, 1.08) | 0.33 (0.07, 1.48) |  | 0.44 (0.31, 0.63) | 0.55 (0.36, 0.85) |
| **Household income**^§^ |  |  |  |  |  |  |  |  |
| Rs 0 to 1000 (Q1) | Ref | Ref |  | Ref | Ref |  | Ref | Ref |
| >1000 to 1900 (Q2) | 0.70 (0.36, 1.38) | 0.68 (0.33, 1.37) |  | 0.70 (0.34, 1.44) | 0.77 (0.36, 1.63) |  | 0.57 (0.41, 0.81) | 0.57 (0.39, 0.83) |
| >1900 to 3000 (Q3) | 0.74 (0.34, 1.58) | 0.77 (0.34, 1.70) |  | 0.85 (0.44, 1.63) | 0.97 (0.49, 1.92) |  | 0.61 (0.42, 0.89) | 0.60 (0.40, 0.89) |
| Rs >3000 (Q4) | 0.84 (0.37, 1.89) | 0.89 (0.38, 2.09) |  | 0.55 (0.27, 1.14) | 0.87 (0.41, 1.85) |  | 0.38 (0.27, 0.54) | 0.54 (0.37, 0.78) |
| Missing (Q0) | ─ | ─ |  | 0.52 (0.14, 1.86) | 0.77 (0.21, 2.90) |  | 0.71 (0.54, 0.95) | 0.83 (0.61, 1.12) |
| **Type of ration card**^†^ |  |  |  |  |  |  |  |  |
| No ration card/ APL | Ref | Ref |  | Ref | Ref |  | Ref | Ref |
| BPL | 1.22 (0.41, 3.64) | 1.09 (0.34, 3.48) |  | 6.55 (1.6, 26.86) | 4.24 (0.96, 18.75) |  | 3.03 (2.42, 3.79) | 2.15 (1.68, 2.75) |
| **CMD** |  |  |  |  |  |  |  |  |
| One | Ref | Ref |  | Ref | Ref |  | Ref | Ref |
| Two | 0.98 (0.52, 1.84) | 0.95 (0.5, 1.8) |  | 0.90 (0.55, 1.5) | 0.94 (0.56, 1.58) |  | 0.80 (0.63, 1.01) | 0.74 (0.57, 0.97) |
| ≥Three | 0.80 (0.30, 2.14) | 0.71 (0.26, 1.96) |  | 0.38 (0.14, 1.06) | 0.32 (0.10, 1.06) |  | 0.72 (0.56, 0.92) | 0.74 (0.56, 0.98) |
| **Health insurance**^†^ |  |  |  |  |  |  |  |  |
| No | Ref | Ref |  | Ref | Ref |  | Ref | Ref |
| Yes | 1.20 (0.65, 2.19) | 1.15 (0.6, 2.18) |  | 2.07 (1.09, 3.96) | 1.29 (0.65, 2.56) |  | 3.14 (2.54, 3.87) | 2.70 (2.17, 3.37) |
| Observation |  | 497 |  |  | 1417 |  |  | 1536 |
| AIC |  | 488 |  |  | 632 |  |  | 1929 |
| BIC |  | 542 |  |  | 706 |  |  | 2004 |

Abbreviations: CMD, cardiometabolic disease; APL, above poverty line; BPL, below poverty line; CI, confidence interval; OR, odds ratio; AOR, adjusted odds ratio; AIC, Akaike's information criteria; BIC, Bayesian information criteria

Data are presented as odds ratios (95% confidence interval)

There were 630 missing observations on household income. These are included in the multivariable logistic regression

^†^2-16 missing observations; ^‡^63 missing observations; ^§^630 missing observations.

There were also 150 missing observations on healthcare providers. These individuals were not included in the analysis.

Multivariable model: Adjusted for all variables listed in that column.

**References**

1. Von Elm E, Altman DG, Egger M, Pocock SJ, Gøtzsche PC and Vandenbroucke JP. Strengthening the Reporting of Observational Studies in Epidemiology (STROBE) statement: guidelines for reporting observational studies. *BMJ*. 2007; 335: 806-8.

2. Nabae K. The health care system in Kerala: its past accomplishments and new challenges. *Natl Inst Public Health*. 2003; 52: 140-5.

3. Wani NUH, Taneja K and Adlakha N. Health system in India: opportunities and challenges for enhancements. *IOSR J Bus Manag*. 2013; 9: 74-82.

4. Keshri V and Ghosh S. Health Insurance for Universal Health Coverage in India: A Critical Analysis based on Coverage, Distribution and Predictors from National Family Health Survey – 4 Data. *BMC Health Serv Res*. 2019: (In review).

5. DataGov India. National Sample Survey (<https://data.gov.in/dataset-group-name/national-sample-survey>). Accessed on March 10, 2022.

6. National Health Authority. Ayushman Bharat: Pradhan Mantri Jan Arogya Yojana (PM-JAY). 2018.

7. World Health Organization. The WHO STEPwise approach to noncommunicable diseases risk factor surveillance: WHO STEPS Surveillance Manual. 2017.
